# Novel Types of Hypermodified Fluorescent Phyllobilins from Breakdown of Chlorophyll in Senescent Leaves of Grapevine (*Vitis vinifera*)

**DOI:** 10.1002/chem.201803128

**Published:** 2018-10-30

**Authors:** Theresia Erhart, Cecilia Mittelberger, Xiujun Liu, Maren Podewitz, Chengjie Li, Gerhard Scherzer, Gertrud Stoll, Josep Valls, Peter Robatscher, Klaus R. Liedl, Michael Oberhuber, Bernhard Kräutler

**Affiliations:** ^1^ Institute of Organic Chemistry & Centre of Molecular Biosciences University of Innsbruck Innrain 80/82 6020 Innsbruck Austria; ^2^ Laimburg Research Centre Laimburg 6-Pfatten (Vadena) 39040 Auer (Ora), BZ Italy; ^3^ Present address: Research Center of Analysis and Test East China University of Science & Technology Meilong Rd 130 200237 Shanghai China; ^4^ Institute of General, Inorganic and Theoretical Chemistry & Centre of, Molecular Biosciences University of Innsbruck Innrain 80/82 6020 Innsbruck Austria; ^5^ Present address: Key Laboratory for Advanced Materials & Institute of, Fine Chemicals, School of Chemistry & Molecular Engineering East China University of Science & Technology Meilong Rd 130 200237 Shanghai China; ^6^ Present address: Faculté des Sciences Pharmaceutiques, Unité de Recherche Enologie EA 4577 Université de Bordeaux 33882 Villenave d'Ornon France

**Keywords:** fluorescence, glycosides, phyllobilin, porphyrinoid, senescence

## Abstract

The tetrapyrrolic chlorophyll catabolites (or phyllobilins, PBs) were analyzed in yellow fall leaves of the grape Chardonnay, a common *Vitis vinifera* white wine cultivar. The major fractions in leaf extracts of *V. vinifera*, tentatively assigned to PBs, were isolated and their structures elucidated. The dominant fraction is a dioxobilin‐type non‐fluorescent Chl‐catabolite of a previously observed type. Two less polar fluorescent PBs were characterized as a novel dioxobilin‐type fluorescent Chl‐catabolite with a bicyclo‐1′,6′‐glycosyl architecture, and its new fluorescent formyloxobilin‐type analogue. The discovery of persistent hypermodified fluorescent PBs with the architecture of bicyclo‐[17.3.1]‐PBs (*bc*PBs), suggests the activity of an unknown enzyme that forges the 20‐membered macroring at the tetrapyrrolic core of a fluorescent PB. *bc*PBs may play specific physiological roles in grapevine plants and represent endogenous anti‐infective agents, as found similarly for other organic bicyclo‐[*n*.3.1]‐1′,6′‐glycosyl derivatives.

## Introduction

**Scheme 1:**
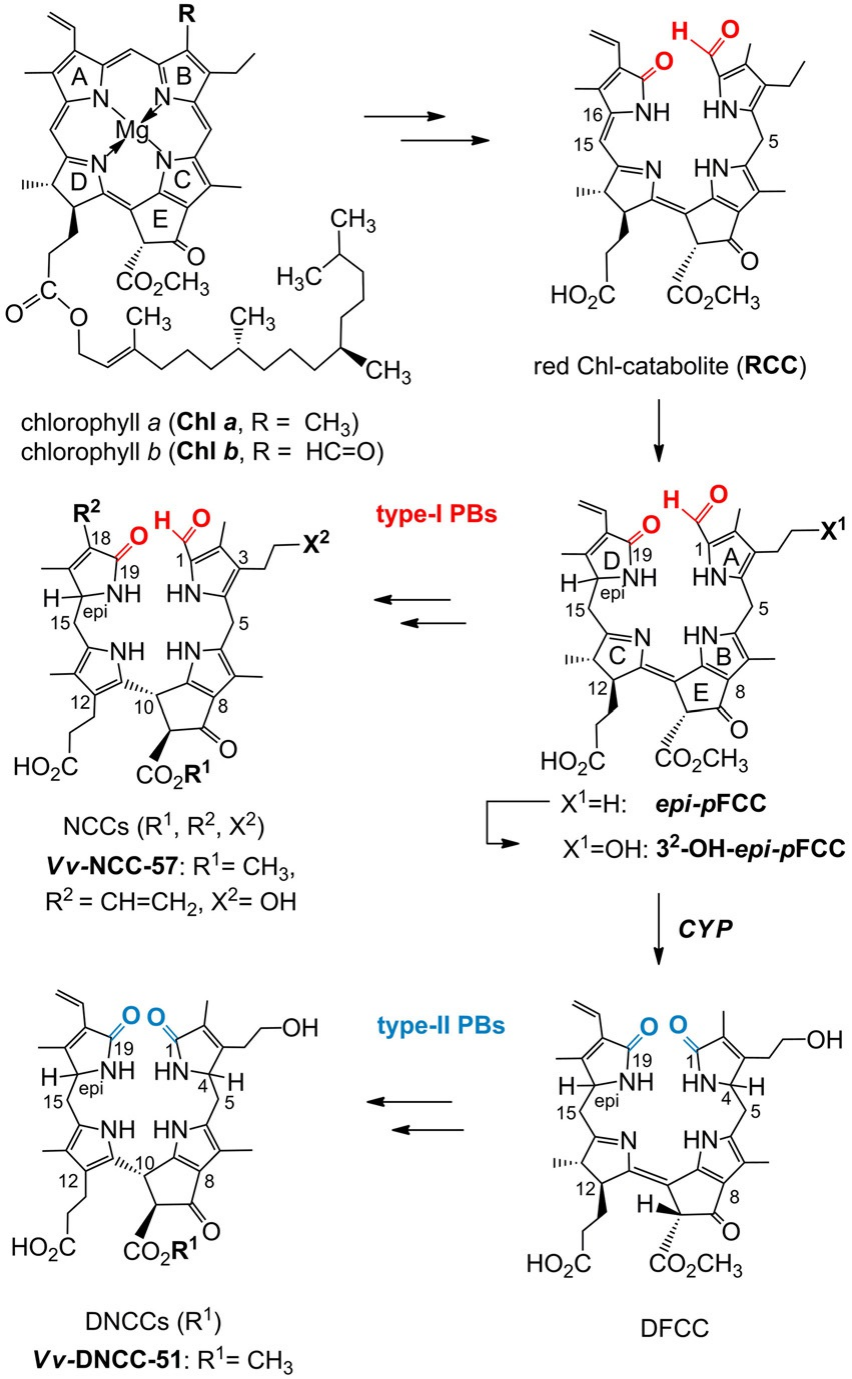
Structural outline of the PaO/phyllobilin pathway of Chl breakdown, presented with key steps of the C16‐stereochemical branch starting with ***epi‐p*** **FCC**. The deformylating enzyme *CYP* (identified as CYP89A9 in *A. thaliana*) converts FCCs (fluorescent type I PBs) into analogous DFCCs (fluorescent type II PBs, the precursors of nonfluorescent DNCCs).

The fall colors of deciduous plants, and the seasonal breakdown of chlorophyll (Chl) in their leaves, have been a stunning biological puzzle until recently.^1^ During the last quarter of a century, breakdown of Chl has been studied intensively in higher plants,1b, 2 and the structures of a large number of tetrapyrrolic Chl catabolites, named phyllobilins (PBs),^3^ were determined in senescent leaves and ripening fruit.2d, 4 Based on complementary chemical and biological investigations, Chl breakdown has been unraveled as following a common PaO/phyllobilin pathway in higher plants (see Scheme 1).2a,2c,2d The first discovered natural PBs were colorless formyloxobilin‐type non‐fluorescent Chl catabolites (NCCs).1b, 2d, 4a, 5 Such colorless tetrapyrroles from Chl degradation accumulate in the vacuoles of senescent leaves.2a There, they are generated by acid‐induced isomerization from their fleetingly existent precursors, the corresponding colorless (formyloxobilin‐type) fluorescent Chl catabolites (FCCs).2a, 6 FCCs arise in the chloroplast in one of two C16‐epimeric forms of “primary” FCCs (*p*FCC or *epi‐p*FCC),^7^ generated by reduction of the red Chl catabolite (RCC)^8^ by a plant‐specific RCC‐reductase (RCCR).2c, 9 The typical further FCC‐functionalization starts with an astounding hydroxylation of *p*FCCs at their saturated 3^2^‐position,1b, 2d catalyzed by the hydroxylase TIC55.^10^ Most of the subsequent further peripheral modifications, derived from the structures of natural FCCs and NCCs, were proposed to occur in the cytosol.2a,2c Indeed, a wide range of colorless non‐fluorescent PBs (NCCs) with one or two peripheral sugar appendages have been characterized,2d, 5, 11 whereas the enzymes involved in the 1′‐glucosylation processes are not yet known.2c

**Scheme 2:**
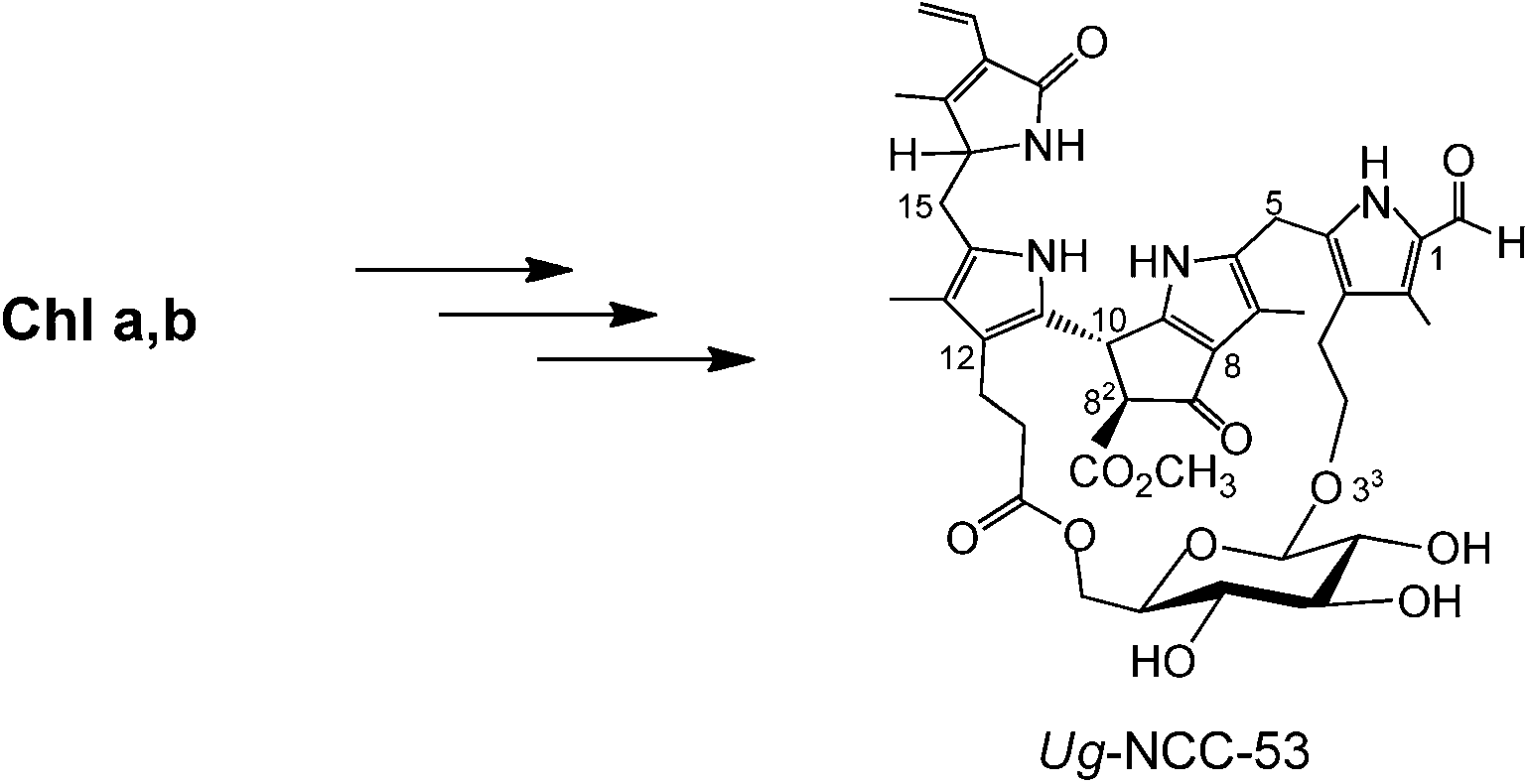
Structural formula of the elm tree bicyclo[17.3.1]‐phyllobilin *Ug*‐NCC‐53.11f

“Hypermodified” fluorescent phyllobilins were first discovered as “hypermodified” FCCs (*hm*FCCs) in ripening bananas (*Musa accuminata*).^12^ The natural *hm*FCCs feature a propionate 6′‐sugar ester, are persistent6b, 13 and give the ripe yellow banana a striking blue glow.12a Related blue luminescent *hm*FCCs were also found in de‐greened leaves of bananas^14^ and other plants.^15^ Furthermore, in senescent leaves of the wych elm tree (*Ulmus glabra*), a natural NCC analogue was recently discovered (named *Ug*‐NCC‐53), in which a d‐glycopyranosyl unit was attached by its C6′ as an NCC propionate ester.11f However, the sugar moiety was bound a second time in this NCC, using a β‐anomeric glysoside bond of C1′ to O3^3^, leading to the intriguing new structural type of a bicyclo[17.3.1]‐phyllobilin (*bc*PB) (see Scheme 2).11f Hence, in this unprecedented *bc*NCC, a bridging 1′,6′‐d‐glycopyranosyl‐linkage was attached twice to the core of an NCC, giving it a remarkably rigidified structure and raising the question of its biosynthetic formation.11f

More recently, an entirely divergent second branch of the PaO/phyllobilin pathway was established that involved colorless dioxobilin‐type non‐fluorescent Chl catabolites (DNCCs).^16^ In *Arabidopsis thaliana*, this path was shown to proceed by the specific isomerization of a corresponding dioxobilin‐type fluorescent Chl catabolite (DFCC).^17^ The crucial divergence, along this pathway, comes about by oxidative de‐formylation of some of the first formed FCCs by a cytochrome P450 enzyme (CYP89A9 in *A. thaliana*),16a generating DFCCs and, thus, providing an entry to the type II PBs (see Scheme 1).

Here, we describe a study of the PBs in extracts of naturally senescent leaves of Chardonnay (*Vitis vinifera*), one of the most important and oldest white wine cultivars worldwide.^18^ In leaf extracts of Chardonnay plants grown in a vineyard in the province of Bozen (northern Italy), we found both, type I and type II PBs, and discovered two remarkably structured new representatives of “hypermodified” fluorescent PBs, as well.2d, 12a The structures of both fluorescent PBs display the exceptional bridging bicyclo[17.3.1]‐1′,6′‐glycopyranosyl architecture, discovered in *Ug*‐NCC‐53.11f This finding helps to specify more closely the pathway of the new branch of Chl breakdown to bicyclo[17.3.1]‐1′,6′‐glycopyranosyl PBs (*bc*PBs) and encourages considering a relevant role of the (fluorescent) *bc*PBs in the endogenous defense against fungal and bacterial pathogens in the grapevine leaves.

## Results

**Figure 1:**
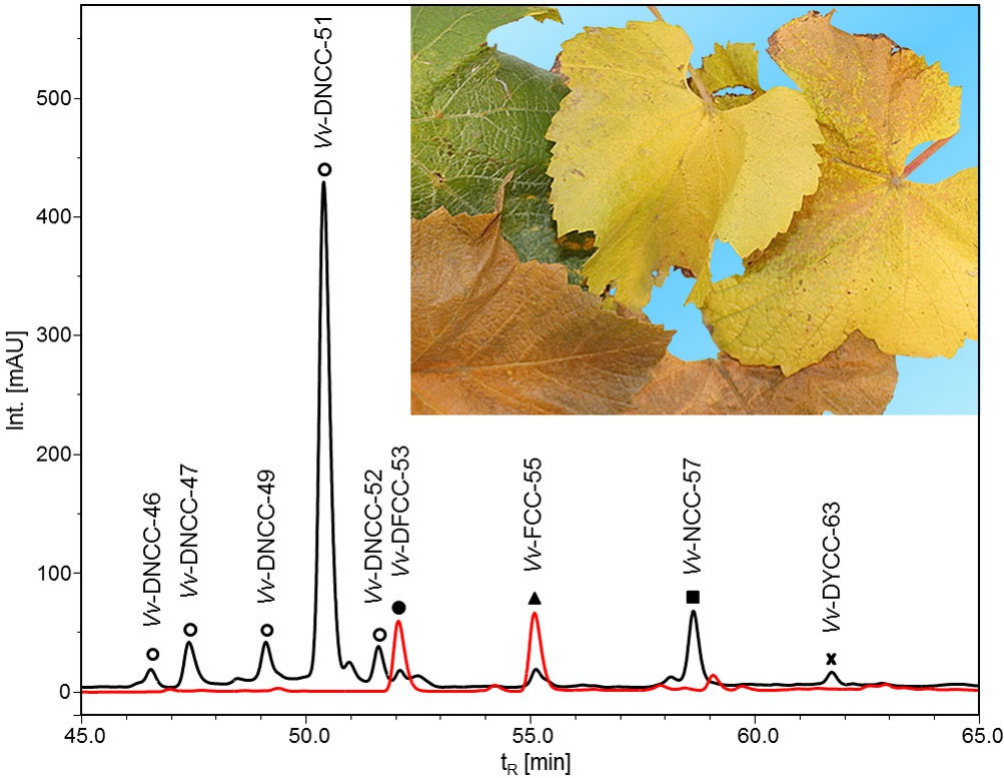
HPLC‐analysis of an extract of senescent Chardonnay leaves with detection of absorption at 250 nm (black trace) and of fluorescence at 450 nm (red trace; see the Experimental Section for details). The inset shows Chardonnay leaves in three different stages of senescence.

Leaves of healthy Chardonnay plants, grown in an experimental vineyard in Piglon, Laimburg, Italy, de‐greened and developed their characteristic golden yellow in late fall of 2014 (see Figure 1). De‐greening of healthy leaves in this region occurs, typically, between the beginning of October and early November and depends on climatic, meteorological and geographical conditions. Yellow, senescent Chardonnay leaves were collected at the experimental vineyard in November 2014, immediately cooled (external ice packages) for transport to the cold‐storage facility, where they were stored at −80 °C, until extraction for further analyses (see the Experimental Section). Extracts of the *V. vinifera* leaves were analyzed by HPLC with UV/Vis and fluorescence detection, leading to the provisional identification of eight colorless PBs and of one yellow PB (see Figure 1). The PBs from *V. vinifera* (*Vv*‐PBs) were tentatively classified as (five) dioxobilin‐type non‐fluorescent Chl catabolites (DNCCs), as a non‐fluorescent (formyloxobilin‐type) Chl catabolite (NCC), as a dioxobilin‐type fluorescent Chl catabolite (DFCC), as a fluorescent (formyloxobilin‐type) Chl catabolite (FCC) and as a dioxobilin‐type yellow Chl catabolite (DYCC). In the extracts of different senescent leaves, the dioxobilin‐type non‐fluorescent Chl catabolite (DNCC) ***Vv*** **‐DNCC‐51** dominated strongly, and the minor *Vv*‐PBs were present in slightly varying relative and absolute amounts. ***Vv*** **‐DNCC‐51** accounted for over 90 % of the *Vv*‐PBs actually isolated from the preparative extract of *V. vinifera* leaves. In the present work, ***Vv*** **‐DNCC‐51**, and four other *Vv*‐PBs, were characterized further, two of which were revealed to represent sugar‐modified fluorescent *bc*PBs, a novel type of Chl catabolites.

**Figure 2:**
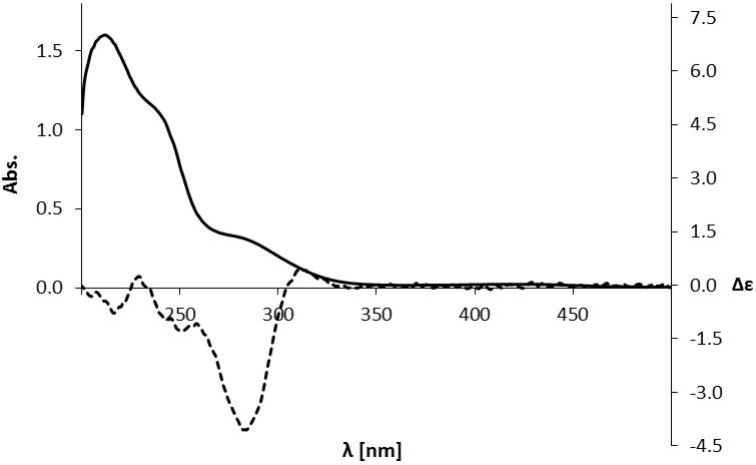
UV/Vis absorption spectrum (solid line) and CD‐spectrum (dashed line, Δ*ϵ* see axis on the right) of Vv‐DNCC‐51 (*c*=3.8×10^−5^ m in MeOH).

**Figure 3:**
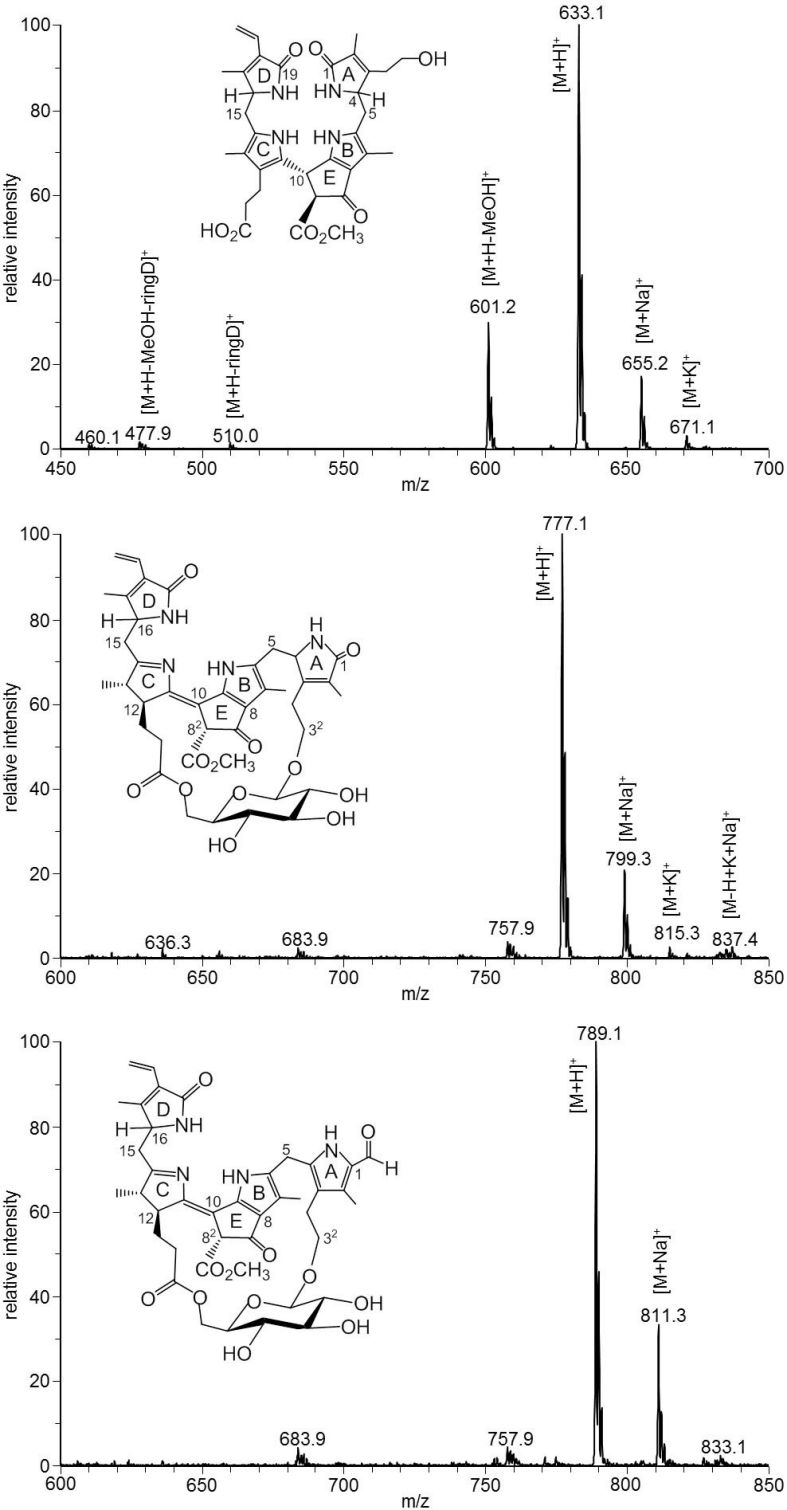
LC/ESI‐MS spectra (positive ions, and assignments) of the *Vv*‐PBs ***Vv*** **‐DNCC‐51** (top), ***Vv*** **‐DFCC‐53** (center) and ***Vv*** **‐FCC‐55** (bottom).

**Figure 4:**
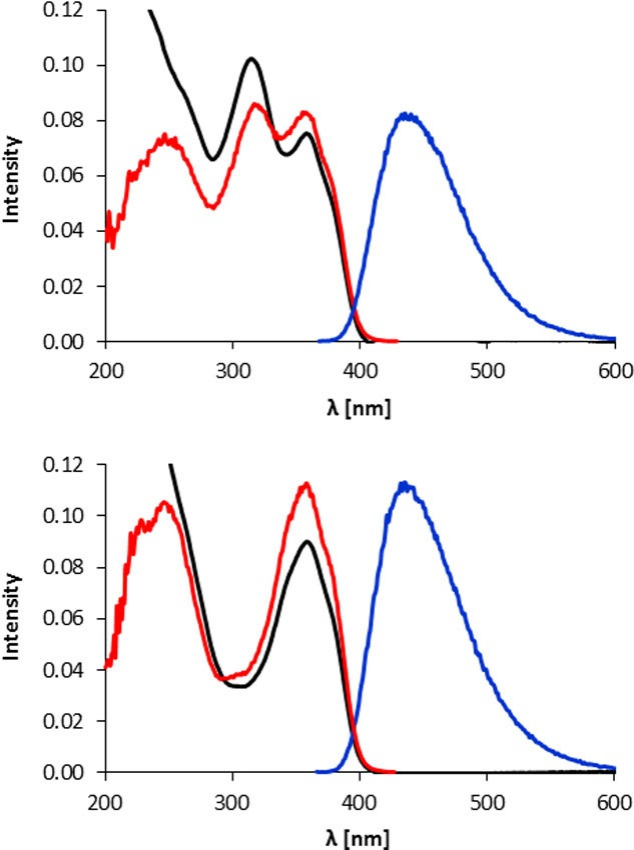
UV/Vis‐absorption spectra, fluorescence emission and fluorescence excitation spectra of ***Vv*** **‐FCC‐53** (top) and of ***Vv*** **‐DFCC‐55** (bottom).

A methanolic extract of 600 g of senescent leaves of *V. vinifera*, from Chardonnay, was separated into fourteen fractions by preparative medium pressure liquid chromatography (MPLC), using the solvent components MeOH and 25 mm aqueous phosphate buffer (pH 7). Two fractions eluting with a nearly 1:1 mixture of the two solvent components contained the major amount of pure ***Vv*** **‐DNCC‐51**, according to analysis by HPLC. Removal of the solvents of these two combined MPLC‐fractions, desalting by the use of SepPak cartridges furnished 60.2 mg (96 μmol) of ***Vv*** **‐DNCC‐51**. From further preparative separation by HPLC of three slightly less polar minor fractions, two blue fluorescent phyllobilins and a nonfluorescent compound (an NCC) were isolated, furnishing pure samples of 0.45 μmol (ca. 0.38 mg) of ***Vv*** **‐DFCC‐53**, 0.78 μmol (0.65 mg) of ***Vv*** **‐FCC‐55** and 1.25 μmol (ca. 0.78 mg) of ***Vv*** **‐NCC‐57**. In an analogous fashion, roughly 0.95 μmol (or 0.59 mg) of a still less polar yellow fraction was isolated as a yellow powder, tentatively identified as a DYCC and named ***Vv*** **‐DYCC‐63**. These five *Vv*‐PB fractions were first classified by their UV/Vis spectra (see Figures 2 and 4, and Supporting Information, Figure S1). They were then further characterized by ESI mass spectra^19^ that furnished their molecular formulas (see Figure 3 and Supporting Information, Figures S2 and S3).

The most polar of the *Vv*‐PBs, named ***Vv*** **‐DNCC‐51** (phenomenologically),^13^ has previously been detected in extracts of *Vv*‐leaves (Pinot noir cultivar), and was suggested to represent a DNCC with the molecular formula C_34_H_40_N_4_O_8_, based on its mass spectrum.16d The ESI‐MS spectrum of the here isolated ***Vv*** **‐DNCC‐51** confirmed the proposed molecular formula (see Figure 3 and Experimental Section). The structure of ***Vv*** **‐DNCC‐51** was fully characterized here, confirming the earlier, tentative, proposal of the chemical constitution of this DNCC. Interestingly, in the extract of *V. vinifera* leaves, four relatively polar PB‐fractions with UV/Vis absorption spectra of a DNCC were observed, isomers (epimers) of ***Vv*** **‐DNCC‐51**, according to their mass spectra, but not characterized further.

In the *V. vinifera* leaf extract, the slightly less polar fraction of a fluorescent *Vv*‐PB was classified as a DFCC based on its UV/Vis absorption spectrum, and named ***Vv*** **‐DFCC‐53** (see Figure 4).^17^ It showed a strong fluorescence, with an emission maximum at 435 nm, and with an excitation spectrum matching its electronic absorption properties (see Figure 4). Analysis of the mass spectrum of ***Vv*** **‐DFCC‐53** revealed a pseudo‐molecular ion [*M*+H]^+^ at *m*/*z* 777.1 (see Figure 3 and Experimental Section). This ion is consistent with a molecular formula of C_40_H_48_O_12_N_4_, suggestive of an exceptional glycosylated type II PB.

The third fraction was named ***Vv*** **‐FCC‐55**, as it had a retention time of about 55 min and was an FCC, according to its UV/Vis‐spectrum (see Figures 1 and 4). Its strong fluorescence had an emission maximum at 440 nm, with a characteristic excitation spectrum of an FCC^20^ (see Figure 4). The ESI mass spectrum of ***Vv*** **‐FCC‐55** revealed a pseudo‐molecular ion [*M*+H]^+^ at *m*/*z* 789.1, consistent with a molecular formula of C_41_H_48_O_12_N_4_ (see Figure 3 and Experimental Section). Hence, ***Vv*** **‐FCC‐55** was indicated to contain one carbon atom more per molecule than ***Vv*** **‐DFCC‐53**, suggesting their close structural relationship.

The ESI mass spectrum of the less polar fraction of ***Vv*** **‐NCC‐57**, classified as an NCC by its UV/Vis‐spectrum (see Experimental Section and Supporting Information, Figure S1) showed a pseudo‐molecular ion [*M*+H]^+^ at *m*/*z* 645.3 (see Experimental Section and Supporting Information, Figure S2), consistent with a molecular formula of C_34_H_40_N_4_O_8._ This indicated ***Vv*** **‐NCC‐57** to contain one carbon atom more per molecule than ***Vv*** **‐DNCC‐51**. HPLC‐analysis, including the co‐injection of ***Vv*** **‐NCC‐57** and of *Cj*‐NCC‐1 (see Experimental Section and Supporting Information, Figure S4), confirmed the identity of ***Vv*** **‐NCC‐57** with an abundant NCC of the “*epi*”‐series, first obtained from senescent leaves of *Cercidiphyllum japonicum,* named *Cj*‐NCC‐1.4b, 6a This established the C16 “*epi*”‐configuration of ***Vv*** **‐NCC‐57** and also indicated the common C16‐configuration as “*epi*” for the other colorless *Vv*‐PBs.

A still less polar yellow fraction was, tentatively, identified as a DYCC by its UV/Vis absorption spectrum and named ***Vv*** **‐DYCC‐63**. It exhibited a strong band at 426 nm in its UV/Vis spectrum, but none near 320 nm, as expected for a yellow dioxobilin‐type (i.e. type II) PB. Its mass spectrum showed a pseudo‐molecular ion [*M*+H]^+^ at *m*/*z* 631.3, consistent with a molecular formula of C_34_H_38_N_4_O_8_ (see Experimental Section and Supporting Information, Figures S1 and S3).

**Figure 5:**
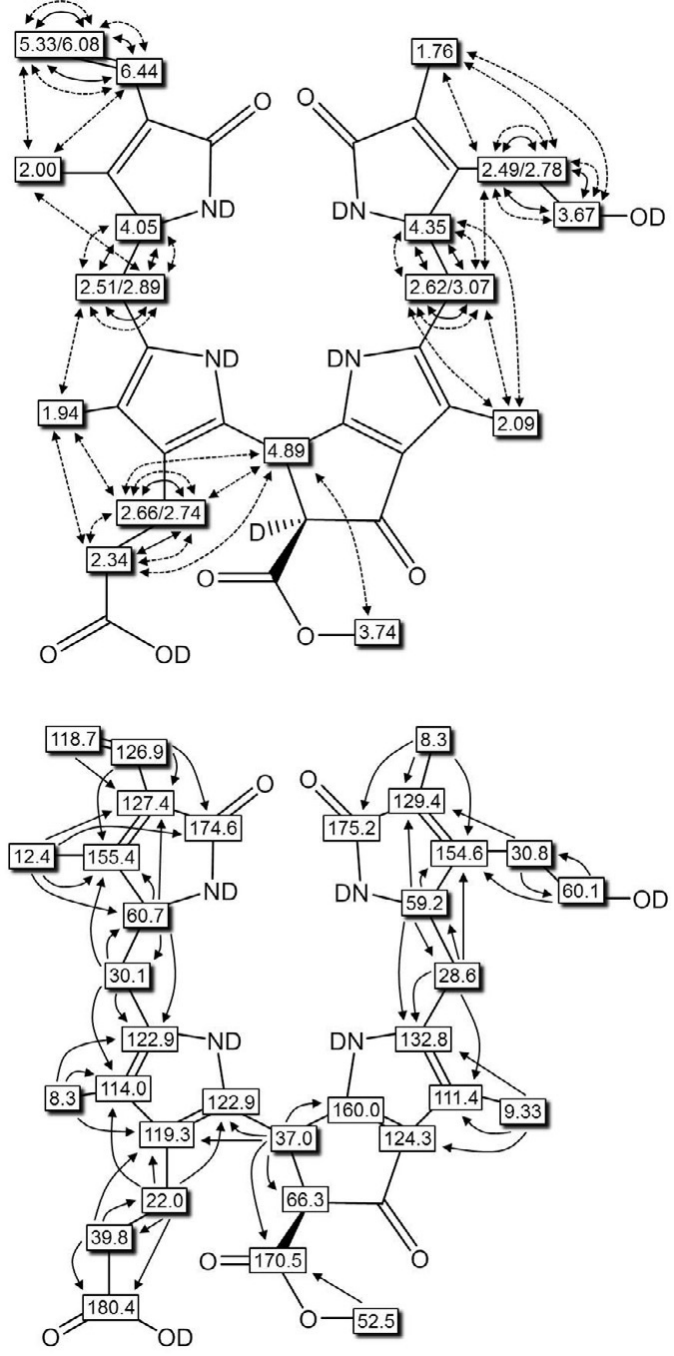
Graphical analysis of NMR data of ***Vv*** **‐DNCC‐51** (500 MHz, CD_3_OD, 12 °C) taken from ^1^H,^1^H‐homonuclear NMR‐spectra (top; full and dotted arrows refer to COSY and ROESY‐correlations, respectively) and ^1^H,^13^C‐heteronuclear HSQC‐ and HMBC‐spectra (bottom; arrows indicate HMBC‐correlations).

The structures of the PBs ***Vv*** **‐DNCC‐51**, ***Vv*** **‐DFCC‐53** and ***Vv*** **‐FCC‐55** were elucidated further by detailed homo‐ and hetero‐nuclear high field NMR analysis. A 500 MHz ^1^H NMR of ***Vv*** **‐DNCC‐51** (in CD_3_OD, at 12 °C) showed resonances of a DNCC substituted with a vinyl group, a methyl ester function and a free propionic acid side chain (see Supporting Information, Figure S5). The signals of all 33 exchange‐stable H‐atoms of ***Vv*** **‐DNCC‐51** were found and assigned, as were 33 of the 34 carbon atoms of this PB (see Experimental Section and Supporting Information, Table S1). From homonuclear ^1^H,^1^H‐ROESY‐ and COSY‐spectra and heteronuclear ^1^H,^13^C‐HSQC and HMBC‐spectra the chemical constitution of ***Vv*** **‐DNCC‐51** could clearly be established (see Figure 5). Its CD‐spectrum (see Figure 2) was consistent with (*R*)‐configuration at C10, establishing the structure of ***Vv*** **‐DNCC‐51** as a 1,4,16,19‐tetrahydro‐16‐*epi*‐1,19‐dioxo‐phyllobilane (see Scheme 1 and Figure 3), the C16 epimer of a DNCC isolated as *At*_MES_‐DNCC‐38 from the *Arabidopsis* MES16 (METHYLESTERASE16)‐ mutant.^21^

In a 700 MHz ^1^H NMR of ***Vv*** **‐DFCC‐53** (in CD_3_OD, at 25 °C) resonances of a vinyl group stood out, of a methyl ester function, and of four methyl groups (three singlets and a doublet) at high field (see Supporting Information, Figure S6). The signals of all 42 non‐exchangeable H‐atoms of ***Vv*** **‐DFCC‐53** were found and assigned, as were the signals of all 40 carbon atoms of this PB (see Experimental Section and Supporting Information, Table S2).

**Figure 6:**
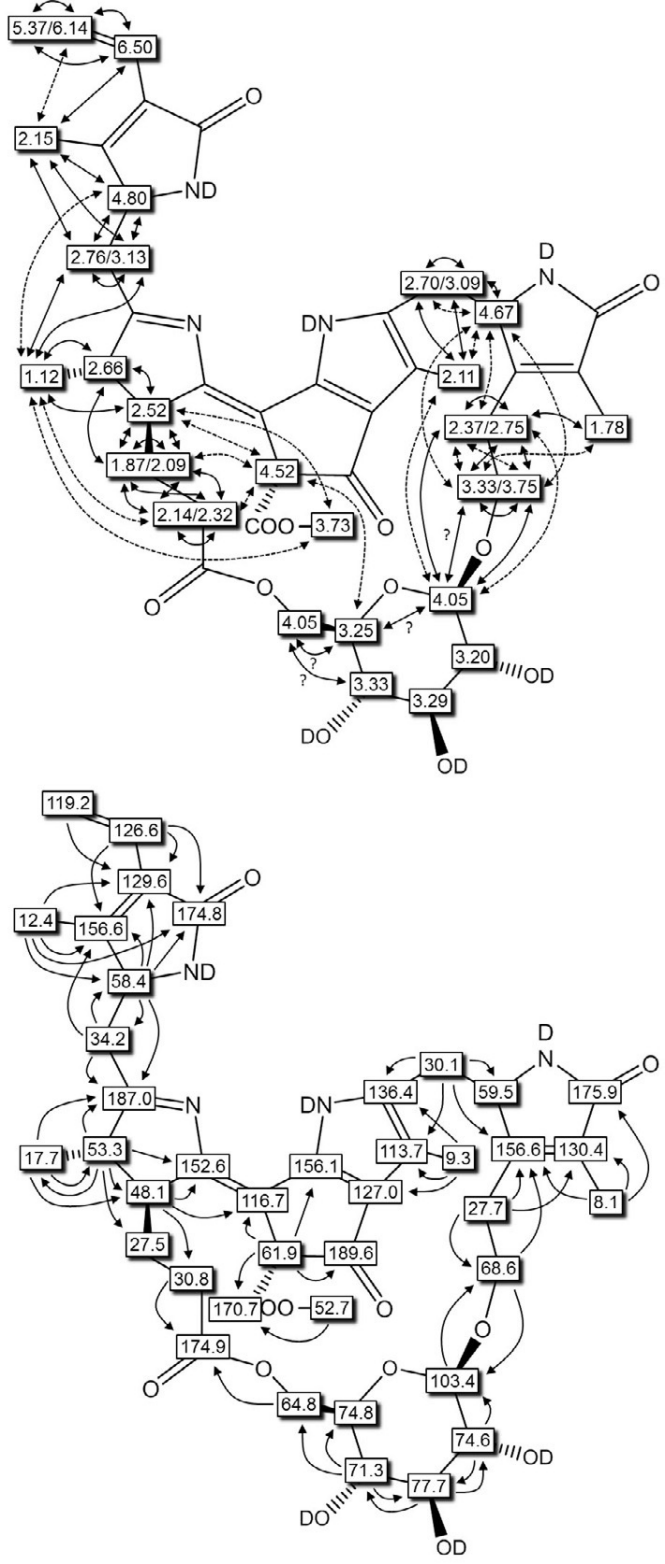
Graphical analysis of the NMR‐data of ***Vv*** **‐DFCC‐53** (600 MHz, CD_3_OD, 0 °C) from ^1^H,^1^H‐ROESY‐spectra (top: full and dotted arrows refer to strong and weaker ROESY‐correlations, respectively; ? indicates ambiguous assignment due to signal overlap) and from ^1^H,^13^C‐ HSQC‐ and HMBC‐spectra (bottom: arrows signify HMBC‐correlations).

**Scheme 3:**
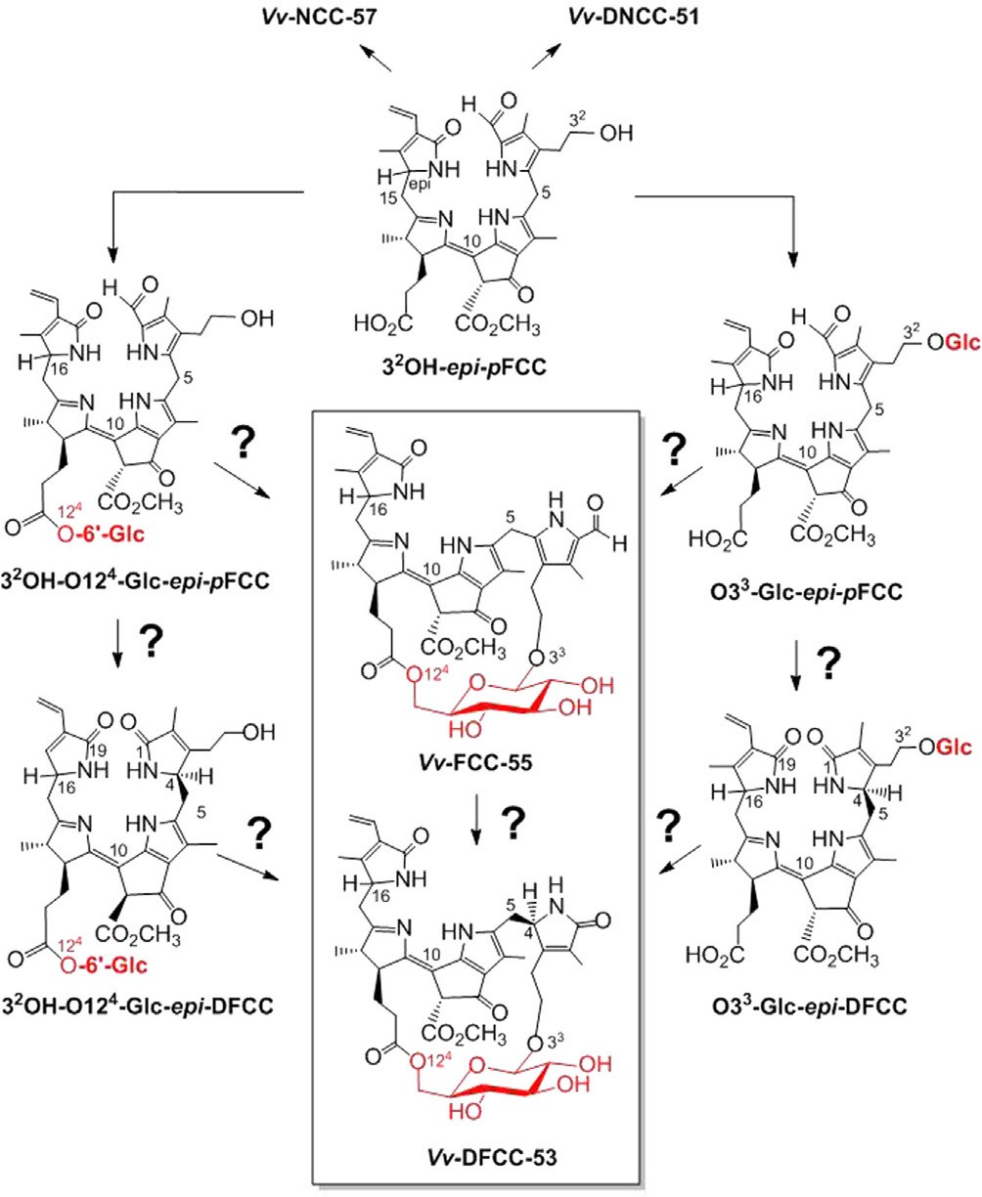
Structural outline of hypothetical biosynthetic paths to the fluorescent *bc*PBs, ***Vv*** **‐DFCC‐53** and ***Vv*** **‐FCC‐55**. Starting from 3^2^‐OH‐*epi‐p*FCC, it may involve FCCs and DFCCs “hypermodified” as 6′‐d‐glucopyranosyl esters (left lane), or modified by alternative β‐d‐glucopyranosylation at their O3^3^ (right lane). Glc: d‐glucopyranose.

A second set of 500 MHz NMR spectra from a solution of ***Vv*** **‐DFCC‐53** in CD_3_CN exhibited the full signal of the exchange labile H‐atom at C8^2^ of ring E (see Supporting Information, Table S4 and Figure S8). The correlation of HC8^2^ to HC5′ of the glucopyranose moiety in the NOE‐spectrum provided evidence for the close mutual positioning of these two units in space. ^1^H,^13^C heteronuclear spectra (in both solvents) provided a set of single bond correlations (HSQC) and multi‐bond correlations (HMBC) that established the two sites of covalent attachment of a pyranose‐unit to O3^3^ of ring A and O12^4^ of the propionate substituent of the PB core of ***Vv*** **‐DFCC‐53**. The ^1^H and ^13^C chemical shifts at the methylene group H_2_C3^2^ were also consistent with an attached peripheral sugar substituent, as were the ^13^C shifts of C5′and C6′ of the sugar moiety with an ester linkage at C6′ (see Figure 6). The bridging sugar‐moiety of ***Vv*** **‐DFCC‐53** was (further) identified as a 1′β‐glycopyranosyl group by comparison of the chemical shifts of its ^1^H and ^13^C atoms with those of the sugar moiety of *Ug*‐NCC‐53.11f Based on the further stereochemical characterization (see below), ***Vv*** **‐DFCC‐53** is deduced to be a 4*R*,8^2^ *R*,12*S*,13*S*,10*Z*,16‐*epi*‐O3^3^,O12^4^‐(1′β,6′‐d‐glycopyranosyl)‐1,19‐dioxo‐1,4,16,19‐tetrahydrophyllobilene‐*b*, that is, a DFCC with a unique and partially rigidified bicyclo‐[17.3.1]‐structure (see Scheme 3).

Likewise, a 700 MHz ^1^H NMR of ***Vv*** **‐FCC‐55** (in CD_3_OD, at 25 °C) showed resonances of a formyl group and of a vinyl group, the singlet of a methyl ester function, and three singlets and a doublet, characteristic of the four other methyl groups of an FCC (see Supporting Information, Figure S7). The signals of all 41 exchange stable H‐atoms of ***Vv*** **‐FCC‐55** were found and assigned, as were 37 of the 41 C‐atoms of this PB (see Experimental Section and Supporting Information, Table S3). A second set of 600 MHz NMR spectra from a solution of ***Vv*** **‐FCC‐55** in CD_3_CN provided complementary data including those of the exchange labile H‐atom at C8^2^ of ring E (see Supporting Information, Table S5 and Figure S9). The NOE‐correlation of HC8^2^ to HC5′ of the glucopyranose moiety in the spectrum of ***Vv*** **‐FCC‐55** provided evidence for the close mutual positioning of these two units and for the indicated macrocyclic structure.

**Figure 7:**
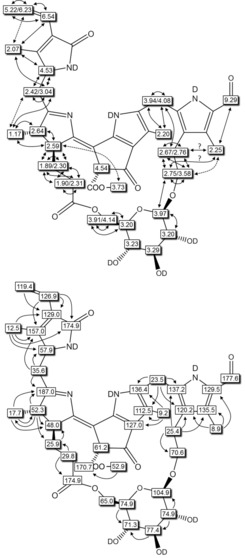
Graphical analysis of the NMR‐data of ***Vv*** **‐FCC‐55** (CD_3_OD, 0 °C) from 500 MHz ^1^H,^1^H‐ROESY‐spectra (top: full and dotted arrows refer to strong and weaker ROESY‐correlations, respectively; ? indicates ambiguous assignment due to signal overlap) and from 600 MHz ^1^H,^13^C‐ HSQC‐ and HMBC‐spectra (bottom: arrows signify HMBC‐correlations).

^1^H,^1^H homonuclear correlations (ROESY‐spectra) and ^1^H,^13^C heteronuclear correlations (HSQC and HMBC spectra) in CD_3_OD solution (see Figure 7) and in CD_3_CN (see Supporting Information, Table S5 and Figure S9) indicated an attachment of a sugar moiety at O3^3^. The shifts of the ^1^H and ^13^C signals for the C3^2^ methylene group were also consistent with the presence of a glycosidic substituent at O3^3^. The chemical shifts of C12^3^ at ring C were consistent with the presence of a propionyl ester functionality and indicated a link to the primary oxygen at C6′ of a glucopyranosyl unit. Likewise, the chemical shifts and heteronuclear correlations of the carbons C5′ and C6′ of the sugar moiety were consistent with the presence of an ester linkage at C6′. Based on the NMR‐spectral data, ***Vv*** **‐FCC‐55** was, thus, assigned the structure of a 8^2^ *R*,12*S*,13*S*,10*Z*,16‐*epi*‐O3^3^,O12^4^‐(1′β,6′‐d‐glycopyranosyl)‐1‐formyl‐19‐oxo‐16,19‐dihydro‐phyllobilene‐*b*, that is, of an FCC connected by O3^3^ and O12^4^ to a 1′β,6′‐glycopyranosyl bridge, and generating a bicyclo‐[17.3.1]‐structure (see Scheme 3).

The collected ^1^H and ^13^C chemical shift data of ***Vv*** **‐DFCC‐53** and of ***Vv*** **‐FCC‐55** (see Supporting Information, Tables S2, S4 and S5) established their closely related structures, which differ merely by the presence of the formyl group at the pyrrole C1 of ***Vv*** **‐FCC‐55**, or its absence at the pyrrolidinone C1 of ***Vv*** **‐DFCC‐53**. Likewise, a comparison of the ^1^H and ^13^C chemical shift data of ***Vv*** **‐FCC‐55** and of *Ug*‐NCC‐53 from senescent wych elm leaves11f (see Experimental Section and Supporting Information, Table S3) was fully consistent with the high structural similarity and the derived isomeric ring B/C‐sections of ***Vv*** **‐FCC‐55** and of *Ug*‐NCC‐53.

**Figure 8:**
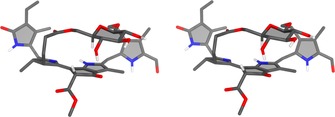
Stereo‐projection of the calculated BP86/def2‐TZVP‐optimized (gas phase) structure of ***Vv*** **‐FCC‐55** (in these calculations the configuration at C16 has arbitrarily been set as R).

Density functional theory (DFT) calculations (BP86/def2‐TZVP) were used to model gas phase structures of ***Vv*** **‐FCC‐55** and ***Vv*** **‐DFCC‐53**. These investigations gave insights into the existence the of ***Vv*** **‐FCC‐55** and ***Vv*** **‐DFCC‐53** as stable PBs. They also supported the derived Lewis formulae, with a short bond between C10 and C11 (calculated bond length of 1.36 Å), as well as an (almost) planar arrangement of the unsaturated system extending over the B/E‐ and C‐ring sections of ***Vv*** **‐FCC‐55 (**with a C10‐C11 dihedral angle of 9°). Unlike the previously studied *Ug*‐NCC‐53,11e the macrocyclic sugar moiety is on the “upper” face of the B/E‐ring of ***Vv*** **‐FCC‐55** (see Figure 8 for the stereo‐projection of a calculated conformer). The mutual arrangement of the glucose and tetrapyrrole moieties are in line, qualitatively, with NOE‐data derived from homonuclear ROESY spectra (see Supporting Information, Figure S9 and S13) with a calculated distance of 2.7 Å between HC8^2^ (of the FCC moiety) and the glucopyranose HC5′. For a qualitative comparison, the structure of *Ug*‐NCC‐53, derived from the molecular dynamics study,11f was also optimized computationally (see Supporting Information, Figure S19), indicating a higher stability, by around 75 kJ mol^−1^, of *Ug*‐NCC‐53, compared to its fluorescent isomer, ***Vv*** **‐FCC‐55**, in the respective calculated conformations.

**Figure 9:**
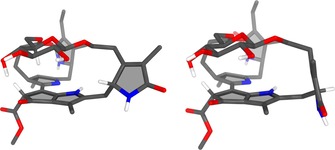
Models of the calculated BP86/def2‐TZVP‐optimized structures of the 4R‐ and 4S‐epimers (left and right, respectively) of ***Vv*** **‐DFCC‐53** (in these calculations the configuration at C16 (which is “*epi*”) has arbitrarily been set as R). Note the pronounced “*endo*” resp. “*exo*” orientation of HC4 in the calculated structures of these two C4‐epimers of ***Vv*** **‐DFCC‐53**.

The (gas phase) structures of both of the C4‐stereoisomers of ***Vv*** **‐DFCC‐53** were also modelled, which differed in the configuration at C4. The sugar moiety was again calculated as sitting “atop” of the B/E‐ring section, positioning the glucose HC5′ at a distance of 2.6 Å from HC8^2^ of the *bc*PB, orienting ring A nearly orthogonal to the B/E‐ring plane, and presenting the C2−C3‐periphery to the top side of both isomeric molecules. Hence, HC4 is pointing towards the glucopyranosyl group (i.e. “endo”) in the *R*‐epimer, but in the opposite direction (i.e. “exo”) in the *S*‐epimer (see Figure 9 and Supporting Information, Figures S15–S18). NOE correlations between HC4 and H_2_C3^2^ are observed in ROESY spectra of ***Vv*** **‐DFCC‐53**, compatible with an “*endo*” position of HC4, as seen in the model of the *R*‐epimer. Interestingly, the quantum chemical studies also revealed *R‐**Vv*** **‐DFCC‐53** to be slightly more stable in the gas phase than its *S*‐epimer (1.7 kJ mol^−1^ or 10.4 kJ mol^−1^, without or with incorporation of dispersion interactions, respectively).

## Discussion and Outlook

Grapevine (*Vitis vinifera*) is a widespread and prehistoric domestic agricultural plant. It is an exceedingly valuable crop worldwide,^22^ with Chardonnay being one of the most important white wine cultivars. Beside their use for wine production, grapes can be sold fresh on markets and in stores, are the basis for juice production, or can be dried as raisins.^18^, ^23^ Additionally, the use of grapevine leaves is popular in different cuisines, especially in Greek, oriental and Asian cooking (see, e.g. 24). In fall, grapevine leaves of white wine cultivars undergo a color change to bright orange, a sign of the seasonal Chl breakdown and leaf senescence.2c

As shown here with the example of leaves of a Chardonnay cultivar, in naturally senescent leaves of grapevine (*V. vinifera*) type I and type II phyllobilins (PBs) accumulate,2d, 3 as was also found in other higher plants, recently.16a, 25 However, the structures of some *Vv*‐PBs reveal a unique pattern of PB‐modifications. Two novel types of fluorescent PBs, ***Vv*** **‐DFCC‐53** and ***Vv*** **‐FCC‐55**, in particular, were discovered here and found to belong to the exceptional class of the bicyclo[17.3.1]‐phyllobilins (*bc*PBs) with a 1′β,6′‐d‐glycopyranosyl bridge linking O12^4^ and O3^3^. Fluorescent *bc*PBs are a special variant of the “hypermodified” FCCs (*hm*FCCs) that are made persistent by an ester modification of their propionate function.6b The two fluorescent *bc*PBs show the amazing structural features actually discovered with the non‐fluorescent analogue *Ug*‐NCC‐53, isolated from senescent leaves of the wych elm.11f The sugar‐bridged bicyclo[17.3.1]‐architecture of *Ug*‐NCC‐53 imposed a rather rigid framework onto the flexible core structure of this NCC, giving it extraordinary 3D‐structural features. The structure of *Ug*‐NCC‐53 encouraged to consider relevant physiological roles for this *bc*NCC and to look out for convincing insights into its biosynthetic formation during Chl breakdown.11f Indeed, the presence of two NCCs glycosylated at their O3^3^ in leaf extracts of the elm tree suggested the occurrence of the corresponding FCCs as catabolic precursors. In spite of this, the specific pathway to the *bc*NCC *Ug*‐NCC‐53 has remained obscure.11f

The existence of the two fluorescent *bc*PBs, ***Vv*** **‐DFCC‐53** and ***Vv*** **‐FCC‐55**, displaying the amazing bicyclo[17.3.1]‐architecture with a 1′6′‐glycopyranosyl‐moiety bridging O12^4^ and O3^3^ in the two novel *Vv*‐PBs, contrasts the presumption that the existence of such bridges would be restricted to the *bc*NCCs, such as *Ug*‐NCC‐53.11f Based on computational modelling, this NCC appeared to be a rather unstrained and stable bicyclo[17.3.1]‐glycosidic molecule, by virtue of the adaptive structure of its flexible phyllobilane backbone.11f Strikingly, as shown here, fluorescent *bc*PBs, with an unsaturated linkage between rings B/E and C, also exist and are generated in the course of the PaO/phyllobilin pathway of Chl breakdown in grapevine leaves.

The occurrence of ***Vv*** **‐DFCC‐53** and ***Vv*** **‐FCC‐55**, in senescent leaves of grapevine, requires the existence of a biosynthetic path to such fluorescent *bc*PBs that generates the key bicyclo[17.3.1]‐structure on the level of fluorescent PBs. In order to analyze this puzzling fact, several biosynthetic sequences could be taken into consideration (see Scheme 3). They would, first, involve one of two basic alternative paths to FCCs with one sugar attached, either of an FCC glucosylated at their O3^3^, or of a “hypermodified” FCC (*hm*FCC) esterified with a d‐glucopyranose at the carboxyl‐O12^4^. Various NCCs with a glucopyranosyl group attached by its anomeric C1′ to an OH‐group (either 3^2^‐OH or 18^2^‐OH) at the periphery are known.2d, 5, 11b The formation of such glucosylated NCCs from breakdown of Chl is believed to proceed by isomerization of the corresponding glucosylated FCC.2a Indeed, the glucosylation pattern of O3^3^‐Glc‐*epi‐p*FCC has precedence in its C16‐epimer (O3^3^‐Glc*‐p*FCC), identified tentatively as *At*_MES_‐FCC‐1 in senescent leaves of a MES16‐deficient *A. thaliana* mutant.^26^ This finding also supported the assumption that the peripheral glucosylations, detected in NCCs, are introduced at the stage of the corresponding FCCs.2a

The structure and glucosylation pattern of 3^2^OH‐O12^4^‐Glc‐*epi‐p*FCC has, likewise, precedence in *hm*FCCs, identified in ripening fruit and in senescent leaves of banana (*M. accuminata*).12a, 27 Two anomeric versions of the *hm*FCC 3^2^OH‐O12^4^‐Glc‐*epi‐p*FCC were found (as *Ma*‐FCC‐63 and *Ma*‐FCC‐64) in extracts of the banana leaves.^27^ Esterification of the propionic acid group of FCCs inhibits their acid‐induced isomerization to the corresponding NCCs. Hence, it makes *hm*FCCs persistent, and *hm*FCCs tend to accumulate in senescent leaves.2d, 6b The corresponding glucose esters of DFCCs, such as the hypothetical 3^2^OH‐O12^4^‐Glc‐*epi*‐DFCC (see Scheme 3), have no precedence among structurally characterized PBs and may not represent intermediary stages of Chl breakdown. However, an isomeric O3^3^‐Glc‐DNCC has been identified, tentatively, in an *A. thaliana* mutant, lacking MES16,^28^ suggesting the fleeting existence of a corresponding DFCC precursor. For either type of sugar‐modified FCC or DFCC, a second biosynthetic step would be required to close the ring to the bicyclo[17.3.1]‐motif.

Indeed, an entirely alternative biosynthetic formation of ***Vv*** **‐DFCC‐53** may proceed directly by oxidative deformylation of its FCC‐precursor, the bicyclo‐glycosylated ***Vv*** **‐FCC‐55**. This scenario would require the corresponding structural tolerance of the deformylating cytochrome P450 enzyme (CYP89A9 in *A. thaliana*)16a for the FCC‐substrates, which may not be likely, but has not been tested yet. ***Vv*** **‐DFCC‐53** represents a single stereoisomer, tentatively assigned here as the 4*R*‐epimer, from comparison of the calculated structures of 4*S*‐ and 4*R*‐epimers of the DFCC with experimental NOE‐correlations.

Glucosylations, as observed in various NCCs,2d, 5 were first interpreted as a typical result of “secondary” metabolism in the context of the “Chl‐detoxification” hypothesis of the Chl breakdown path.^29^ However, the formation of *hm*FCCs in ripening fruit and senescent leaves appeared to be a rational consequence of a deliberate “biosynthetic” effort of some plants, with the purpose of generating luminescent pigments.^12^, ^14^ The discovery of the fluorescent *bc*PBs ***Vv*** **‐FCC‐55** and ***Vv*** **‐DFCC‐53** in grapevine leaves, likewise, suggests a special “biosynthetic” input in generating such “persistent” *bc*PBs, rationalized, again, by a physiological benefit in the leaves from such *bc*PBs.

So far, the 1′‐β‐d‐glycosyl‐transferases involved in the formation of the FCC‐glucosides, such as O3^3^‐β‐d‐Glc*‐p*FCC,^26^ are unknown,2a as are plant acyltransferases to sugars^30^ of a type required for the assembly of *hm*FCCs2d from an activated FCC. Hence, both types of enzymes remain to be identified in higher plants. Likewise, unknown are the plant enzymes capable of forging the 20‐membered macro‐ring in the bicyclo‐1′,6′‐glycosyl‐architecture of *bc*PBs by setting up the second one of the two conjugations of the glycopyranosyl linker with the tetrapyrrolic core of an FCC. Along with the current evidence about the location of typical Chl‐catabolic enzymes,2c not only the still elusive enzymes that introduce sugar units in fluorescent PBs, but also those closing the bicyclo‐1′,6′‐glycosyl‐macroring, would be proposed to be active cytosolic proteins.2a This hypothesis would exclude a path to the bicyclo‐1′,6′‐glycosyl‐structure at the stage of an NCC, i.e., after import into the vacuole.11f Clearly, the here reported discovery of the amazing fluorescent *bc*PBs poses intriguing new questions with respect to the biosynthetic paths to these unique Chl‐catabolites with a bicyclo‐1′,6′‐glycosyl‐macroring.

Natural heterocyclic products displaying a sugar bridged bicyclo‐[n.3.1]‐structure were unknown before the discovery of *Ug*‐NCC‐53.11f Typical sugar appendages in natural products are bound as terminal 1′‐glycosides or in a linear oligosaccharide topology.30b, 31 However, the biological toolbox with sugar appendages is far from being explored, and Nature's capacity for “natural‐product glycoengineering” is enormous.30b A range of natural, and semisynthetic non‐pyrrolic organic compounds exhibit the exceptional 1,6‐glycopyranosyl‐bridged macrocyclic bicyclo‐[*n*.3.1]‐structure and are the target of considerable interest from biological and pharmaceutical points of view.11f, 32 Indeed, among such 1,6‐glycopyranose‐bridged organic compounds, also classified as ansaglycosides,32d figure inhibitors of cell growth,^33^ as well as compounds with antifungal,^34^ antibacterial32a, 34b, 35 and antiviral effects.32b, 36 The exceptional *bc*PBs in grapevine leaves may be surmised to play crucial (however, still elusive) physiological roles, both in plants and in humans. Important experimental evidence along these lines comes from the recently identified PBs in pathogenically de‐greened apple and apricot leaves,^37^ suggesting a role for PBs in the interaction of the plant host with bacterial or fungal pathogens, either as part of the plant's immune response^38^ or the pathogen's virulence strategy. Likewise, the possible health effects of such intriguing natural products as components of our daily nutrition are also an attractive, but still unexplored area of research.2d Complex macrocyclic skeletons, like that of *bc*PBs, are a feature of physiologically active natural products, and are recognized as evolutionary privileged structures in modern drug design approaches.32c, 39

Two of the colorless phyllobilins in naturally senescent, golden‐yellow leaves of grapevine (*V. vinifera*) were characterized as an FCC and a DFCC that belong to the wider class of the hypermodified fluorescent PBs and represent the specific subtype of the *bc*PBs with a bicyclo[17.3.1]‐glycosyl structure (see Scheme 3). The biosynthetic generation of this structural feature of *bc*PBs is puzzling, and is a challenge to be pursued further. Comparison of the NOE correlations observed in ROESY spectra of ***Vv*** **‐DFCC‐53** provides evidence in support of the *R*‐configuration at C4, leading to a first tentative stereochemical assignment of a type II PB at the new asymmetric C4. It will be of interest to find further support for the (general) validity of this stereochemical assignment in type II PBs.

The discovery of *bc*PBs, furthermore, not only enlarges the portfolio of the known PBs, and their structural diversity, but may also open a new chapter in the search for the still elusive roles of Chl catabolites in senescent leaves and other plant organs.2a,2d, 4d, 12b Persistent blue fluorescent PBs, such as those now found in grapevine leaves, are (potential) endogenous sensitizers for the formation of singlet oxygen.^20^ They also contribute as natural optical brighteners to the optical appearance of the leaves,^14^ a factor considered relevant in bio‐communication.^40^ The exceptional structures of *bc*PBs may also provide a new drive to the quest for uncovering relevant pharmacological effects of the abundant, and often, uniquely structured PBs.

## Experimental Section

### Plant material

Senescent, yellow colored leaves were collected on November 14th, 2014 from healthy Chardonnay grapevine plants in an experimental vineyard (“Piglon”), at Laimburg Research Centre (Pfatten/Vadena, South Tyrol, Italy). The grapevines, planted in 2006, were grown on a Guyot training system and managed according to the integrated production guidelines. The leaves were transported on ice to the laboratory, immediately frozen to −80 °C, and transported frozen to Innsbruck, where they were stored at −80 °C until analyses.

### Chemicals

HPLC grade methanol (MeOH) and dichloromethane (MeCl_2_) from VWR Chemicals (Vienna, AT); MeCl_2_ was redistilled prior to use; potassium dihydrogen phosphate *puriss. p.a*., potassium phosphate dibasic‐anhydrous *puriss. p.a.,* Sigma–Aldrich (Buchs, CH) and ammonium acetate *puriss. p.a*., from Fluka (Buchs, CH); ultrapure water (18 MΩ cm^−1^) from a Millipore apparatus; SepPak C18 cartridges (1 and 5 g sizes) were from Waters Associates (Milford, USA). The pH values were measured with a WTW Sentix 21 electrode connected to a WTW pH535 digital pH‐meter.

### Methods

**Analytical HPLC**: Dionex UltiMate 3000 HPLC system, UltiMate 3000 pump, UltiMate 3000 diode array detector and RF2000 fluorescence detector, 200 μL injection loop. Phenomenex Hyperclone ODS 5 μm 250×4.6 mm i.d. column protected with a Phenomenex ODS 4×3 mm i.d. pre‐column; flow‐rate 0.5 mL min^−1^. Solvent A: 50 mm aq. potassium phosphate (pH 7), solvent B: MeOH; solvent C: water; solvent composition A/B/C: 0–5 min: 80/20/0; 5–60 min: 80/20/0 to 40/60/0; 60–80 min: 40/60/0 to 0/100/0; 80–85 min: 0/100/0; 85–87 min: 0/20/80; 87–90 min: 80/20/0. Data were collected and processed with Chromeleon V6.80.

**MPLC**: Crude preparative separation of the raw leaf extract. Two Büchi pumps C605, a pump manager Büchi C615, a Büchi detector C635 (detection at 250 nm); home built column (25 cm length, 4 cm diameter), filled with 100 g of 50 μm RP‐18 powder (Sepra C18‐E), provided by Phenomenex. Solvent A: 25 mm aqueous potassium phosphate (pH 7) solvent B: MeOH; solvent composition A/B: 0–5 min: 80/20; 5–70 min: 80/20 to 45/55; 70–110 min: 45/55 to 40/60; 110–160 min: 40/60 to 10/90.

**Semipreparative HPLC**: Dionex UltiMate 3000 HPLC system, UltiMate 3000 pump, UltiMate 3000 diode array detector, 1.13 mL injection loop; 00G‐4252‐NO Luna 5u C18(2) 100A column (250×10 mm i.d.) at 20 °C; column protected with a Phenomenex ODS 4×3 mm i.d. pre‐column; flow‐rate 0.5 mL min^−1^. Solvent A: 50 mm aq. potassium phosphate (pH 7), solvent B: MeOH; solvent C: water; solvent composition A/B/C: 0–5 min: 80/20/0; 5–60 min: 80/20/0 to 40/60/0; 60–80 min: 40/60/0 to 0/100/0; 80–85 min: 0/100/0; 85–87 min: 0/20/80; 87–90 min: 80/20/0. Data were collected and processed with Chromeleon V6.80.

**Desalting by the use of SepPak cartridges**: Raw *Vv*‐PB‐samples, dissolved in potassium phosphate buffers (pH 7) containing residual MeOH (from chromatography) were applied to the cartridge, washed with 20 mL (5 g cartridge) or 5 mL (1 g cartridge) of water and eluted with 20 mL (5 g cartridge) or 5 mL (1 g cartridge) of MeOH.

### Spectroscopy

UV/Vis: Agilent Cary 60 spectrophotometer; *λ*_max_ [nm] (log *ϵ* or relative *ϵ* (*ϵ*_rel_)). CD: Jasco J715, *λ*_min/max_ [nm] (Δ*ϵ*). NMR: Bruker Avance 4 Neo 700 MHz, BrukerUltraShield Avance II+600 MHz or Varian Unity Inova 500 MHz spectrometers; *δ* [ppm], J [Hz], internal references: *δ*(C^*1*^ *H*D_2_OD) 3.31 ppm and *δ*(^*13*^ *C*D_3_OD) 49.0 ppm;^41^ ^13^C‐signal assignment deduced form HSQC and HMBC spectra. Mass spectrometry: Finnigan LCQ Classic, positive ion‐mode, electrospray ionization (ESI) source, 4.5 kV spray voltage; *m*/*z* (rel. abundance, type of ion).

### Leaf extraction and isolation of *Vv*‐PBs

A sample of 600 g (wet weight) of yellow Chardonnay leaves (collected November 14th, 2014), frozen at −80 °C, was crushed cold to a powder with a mechanical mixer, suspended in 500 mL of cold MeOH and again mixed for two more min. The suspension was filtered through a coarse glass filter and the filter cake was washed with 100 mL MeOH. The combined filtrates were stored at 4 °C. The remaining filter cake was re‐suspended in 500 mL of MeOH by mixing mechanically for 2 min and filtered again. This operation was repeated once more. The three filtrates were combined (about 1500 mL) and solvents were removed under vacuum and at <30 °C to a residual volume of roughly 100 mL by the use of a rotatory evaporator. The raw isolate was frozen at −20 °C for overnight storage. Subsequently it was mixed with 1 L of 25 mm aqueous potassium phosphate buffer (pH 5.2) and transferred into a separation funnel, to be extracted by four sequential batches of MeCl_2_ (1 L, 750 mL, 500 mL and 500 mL). The combined organic phases were dried by passage through a large plug of dried cotton wool and the solvents were removed completely under vacuum by the use of a rotatory evaporator. The residue was dissolved in 20 mL of MeOH and 80 mL of 25 mm aqueous potassium phosphate buffer (pH 7) were added. A yellow powder formed, which was removed by centrifugation. The clear supernatant was stored overnight at −80 °C. The sample was applied to the column of the MPLC‐system and was separated into 20 fractions, which were analyzed by HPLC. Fractions 6 and 7 contained pure ***Vv*** **‐DNCC‐51** and were combined (90 mL, in total) and concentrated to about 50 mL containing “pre‐purified” ***Vv*** **‐DNCC‐51**. Fractions 10 and 11, which contained impure ***Vv*** **‐DFCC‐53** (in about 40 mL solvent, each), were also concentrated under vacuum and at <30 °C to a residual volume of roughly 20 mL by the use of a rotatory evaporator, desalted (1 g SepPak cartridge) and stored frozen at −20 °C (as “raw” ***Vv*** **‐DFCC‐53**) for further purification by preparative HPLC (see below). Fractions 12 and 13, which contained impure ***Vv*** **‐FCC‐55** in about 40 mL solvent (each), were also concentrated under vacuum and at <30 °C to a residual volume of roughly 20 mL using a rotatory evaporator and desalted on a 1 g SepPak cartridge. Solvents were removed to furnish two samples of “raw” ***Vv*** **‐FCC‐55** for further purification by preparative HPLC (see below).

**Isolation of** ***Vv*** **‐DNCC‐51**: the sample of “pre‐purified” ***Vv*** **‐DNCC‐51** was used in two similarly sized batches, which were each desalted using a 5 g SepPak C18‐cartrige. Solvents were removed under vacuum and at <30 °C to a residual volume of roughly 2 mL by the use of a rotatory evaporator. The two residual samples were combined and frozen with liq. N_2_ and lyophilized overnight, furnishing 60.2 mg of ***Vv*** **‐DNCC‐51** as an off‐white powder.

**Isolation of** ***Vv*** **‐DFCC‐53**: the two desalted MPLC‐fractions were combined and dissolved in roughly 0.5 mL of a 4:1 (v/v) mixture of 25 mm aqueous phosphate buffer (pH 7) and MeOH for separation by semi‐preparative HPLC in two similarly sized batches. From each run the main fraction (***Vv*** **‐DFCC‐53**) was collected and analyzed by HPLC for purity. The combined purified samples were desalted by the use of a 1 g SepPak cartridge. Solvents were removed using a rotatory evaporator under vacuum and at <30 °C. The residual sample of ***Vv*** **‐DFCC‐53** was dissolved in 20 mL of MeOH and analyzed quantitatively as 0.38 mg by recording its UV/Vis spectrum. Solvents were removed and the residue of ***Vv*** **‐DFCC‐53** was dried using high vacuum, for storage at −80 °C for further analysis.

**Isolation of** ***Vv*** **‐FCC‐55**: The two samples of “raw” ***Vv*** **‐FCC‐55** were dissolved in roughly 0.5 mL each of a 4:1 (v/v) mixture of 25 mm aqueous phosphate buffer (pH 7) and MeOH and separated by semi‐preparative HPLC. The fractions collected were analysed by HPLC for content. From each run the main fraction with pure ***Vv*** **‐FCC‐55** was desalted by the use of a 1 g SepPak cartridge. Solvents were removed using a rotatory evaporator under vacuum and at <30 °C. The dried samples of ***Vv*** **‐FCC‐55** were dissolved in 20 mL of MeOH each and analysed quantitatively as 0.26 mg (from MPLC‐fraction 11) and 0.39 mg (from MPLC‐fraction 12) by recording UV/Vis spectra. The samples of ***Vv*** **‐FCC‐55** were combined, solvent was removed and the residual samples of ***Vv*** **‐FCC‐55** were dried and stored frozen at −80 °C for further analysis.

**Isolation of** ***Vv*** **‐NCC‐57 and of** ***Vv*** **‐DYCC‐63**: from the semi‐preparative HPLC experiments fractions were collected, combined and desalted that had HPLC‐retention times and UV/Vis‐spectral properties of ***Vv*** **‐NCC‐57** or of ***Vv*** **‐DYCC‐63**. The resulting samples of ***Vv*** **‐NCC‐57** and ***Vv*** **‐DYCC‐63** were each dissolved in 5 mL of MeOH and analysed quantitatively by recording their UV/Vis‐spectra, indicating 1.25 μmol (0.78 mg) of ***Vv*** **‐NCC‐57** and 0.95 μmol (0.59 mg) of ***Vv*** **‐DYCC‐63**. Solvents were removed and the residual samples of ***Vv*** **‐NCC‐57** and ***Vv*** **‐DYCC‐63** were dried and stored frozen at −80 °C for mass spectrometric analysis.

### Computational methodology

The initial structure of ***Vv*** **‐FCC‐55** was developed from the previously published NCC11f gas phase structure and modified to be consistent with the stereochemistry derived from NMR (NOE) data using GaussView 6.0^42^ and Schrödinger's Maestro^43^ tool. Subsequently, the initial ***Vv*** **‐FCC‐55** conformer was structurally optimized in the gas phase using Density Functional Theory. The BP86^44^ density functional was employed in combination with the resolution‐of‐identity technique^45^ and the def2‐TZVP basis set.^46^ Empirical dispersion corrections of the Grimme type were also tested^47^ but had little effect on the resulting structures (see overlay in Figure S14) The gas phase structures of the two ***Vv*** **‐DFCC‐53** C4‐stereoisomers were generated from the optimized ***Vv*** **‐FCC‐55** structure. All calculations were performed with Turbomole^48^ and structures were visualized with PyMol.^49^

### Spectroanalytical data

***Vv‐*** **DNCC‐51**: UV/Vis (MeOH, *c*=3.8×10^−5^ m) *λ*_max_ (*ϵ*_rel_)=288 sh (0.26), 241 sh (1.00), 212 (1.49) nm. CD (MeOH, *c*=3.8×10^−5^ m) (Δ*ϵ*): *λ*_max_=323 (0.2), 284 (−4.1), 258 (−1.1), 251 (−1.3), 229 (0.2), 216 nm^−1^ (−0.8) (see Figure 2). ^1^H‐NMR (500 MHz, CD_3_OD, 12 °C): *δ*=1.76 (s, 3 H, H_3_C‐2^1^), 1.94 (s, 3 H, H_3_C‐13^1^), 2.00 (s, 3 H, H_3_C‐17^1^), 2.09 (s, 3 H, H_3_C‐7^1^), 2.26–2.41 (m, 2 H, H_2_C‐12^2^), 2.47–2.54 (m, H_A_C‐3^1^, H_A_C‐15, in total 2 H), 2.59–2.68 (m, H_A_C‐5, H_A_C‐12^1^, in total 2 H), 2.71–2.81 (m, H_B_C‐12^1^, H_B_C‐3^1^, in total 2 H), 2.89 (dd, 1 H, *J*=5.0/14.6, H_B_C‐15), 3.07 (dd, 1 H, *J*=5.0/14.8, H_B_C‐5), 3.61–3.71 (m, 2 H, H_2_C‐3^2^), 3.74 (s, 3 H, H_3_C‐8^5^), 4.05 (dd, 1 H, *J*=4.9/8.5, HC‐16), 4.35 (triplettoid, 1 H, *J*=5.6 Hz, HC‐4), 4.89 (s, 1 H, HC‐10), 5.33 (dd, 1 H, *J*=2.2/11.7, H_A_C‐18^2^), 6.08 (dd, 1 H, *J*=2.2/17.8, H_B_C‐18^2^), 6.44 ppm (dd, 1 H, *J*=11.7/17.8 Hz, HC‐18^1^) (see Supporting Information, Figure S5).^13^C‐NMR data: see Supporting Information, Table S1. ESI‐MS (LCQ) *m*/*z* (%)=1287.1 (18, [2 *M*+Na]^+^); 1265.1 (25, [2 *M*+H]^+^); 655.2 (17, [*M*+Na]^+^); 635.1 (9), 634.1 (41), 633.1 (100, C_34_H_41_N_4_O_8_^+^, [*M*+H]^+^); 601.2 (30, [*M*‐CH_3_OH+H]^+^).

***Vv‐*** **DFCC‐53**: UV/Vis (MeOH, *c*=6.6×10^−5^ m) *λ*_max_ (*ϵ*_rel_)=379 sh (0.64), 357 (1.00), 263 sh (1.02), 243 sh (1.54). CD (MeOH, *c*=6.6×10^−5^ m) (Δ*ϵ*): *λ*_max_=383 sh (0.4), 303 (0.8), 251 (−0.5), 228 nm^−1^ (−0.5) (see Supporting Information, Figure S11). ^1^H‐NMR (600 MHz, CD_3_OD, 0 °C): *δ*=1.12 (d, 3 H, *J*=7.3 Hz, H_3_C‐13^1^), 1.78 (s, 3 H, H_3_C‐2^1^), 1.84–1.90 (m, 1 H, H_A_C‐12^1^), 2.08–2.17 (m, H_B_C‐12^1^, H_A_C‐12^2^, superimposed by 2.11 (s, H_3_C‐7^1^) and 2.15 (s, H_3_C‐17^1^), in total 8 H), 2.29–2.34 (m, 1 H, H_B_C‐12^2^), 2.36–2.41 (m, 1 H, H_A_C‐3^1^), 2.51–2.54 (m, 1 H, HC‐12), 2.66 (q, 1 H, *J*=7.3/14.4 Hz, HC‐13), 2.69–2.79 (m, (H_B_C‐3^1^, H_A_C‐15, superimposed by 2.70 (dd, *J*=9.4/14.6 Hz, H_A_C‐5), in total 3 H), 3.09 (dd, 1 H, *J*=4.9/14.6 Hz, H_B_C‐5), 3.14 (dd, 1 H, *J*=5.2/17.0 Hz, H_B_C‐15), 3.17–3.22 (m, 1 H, HC‐2′), 3.25–3.30 (m, HC‐5′, HC‐3′, in total 2 H), 3.32–3.37 (m, H_A_C‐3^2^, HC‐4′, in total 2 H), 3.71–3.76 (m, H_B_C‐3^2^, superimposed by 3.73 (s, H_3_C‐8^5^), in total 4 H), 4.05 (d, 2 H, *J*=4.1 Hz, H_2_C‐6′), 4.07 (d, 1 H, *J*=7.8 Hz, HC‐1′), 4.52 (s, HC‐8^2^, ca. 0.5 H), 4.64–4.68 (m, 1 H, HC‐4), 4.80 (t, 1 H, *J*=5.6 Hz, HC‐16), 5.37 (dd, 1 H, *J*=2.2/11.7 Hz, H_A_C‐18^2^), 6.14 (dd, 1 H, *J*=2.2/17.7 Hz, H_B_C‐18^2^), 6.50 ppm (dd, 1 H, *J*=11.7/17.7 Hz, HC‐18^1^) (see Supporting Information, Figure S6). ^**13**^ **C‐NMR** data: see Supporting Information, Table S2. **ESI‐MS** (LCQ): *m*/*z* (%)=799.3 (21, [*M*+Na]^+^); 779.2 (14), 778.2 (49), 777.1 (100, C_40_H_49_O_12_N_4_^+^, [*M*+H]^+^). **Fluorescence** (MeOH, *c*=5.3×10^−6^ m): emission spectrum (*λ*_Ex_=358 nm): *λ*_max_=435 nm; excitation spectrum (*λ*_em_=435 nm): *λ*_max_=358 nm.

***Vv*** **‐FCC‐55**: UV/Vis (MeOH, *c*=5.3×10^−5^ m) *λ*_max_ (*ϵ*_rel_)=379 sh (0.37), 356 (0.75), 314 (1.00), 264.0 sh (0.65), 244 sh (0.80). CD (MeOH, *c*=5.3×10^−5^ m) (Δ*ϵ*): *λ*_max_=340 (−1.2), 282 (1.9), 250 (0.1), 241 (0.5), 218 nm^−1^ (−0.4) (see Supporting Information, Figure S12). ^1^H‐NMR (600 MHz, CD_3_OD, 0 °C): *δ*=1.17 (d, 3 H, *J*=7.3 Hz, H_3_C‐13^1^), 1.88–1.93 (m, H_A_C‐12^1^, H_A_C‐12^2^, in total 2 H), 2.07 (s, 3 H, H_3_C‐17^1^), 2.20 (s, 3 H, H_3_C‐7^1^), 2.25 (s, 3 H, H_3_C‐2^1^), 2.28–2.31 (m, H_B_C‐12^1^, H_B_C‐12^2^, in total 2 H), 2.40–2.45 (dd, 1 H, *J*=8.9/18.0 Hz, H_A_C‐15), 2.58–2.61 (m, 1 H, HC‐12), 2.64 (dd, 1 H, *J*=2.6/7.3 Hz, HC‐13), 2.65–2.70 (m, 1 H, H_A_C‐3^1^), 2.73–2.83 (m, H_A_C‐3^2^, H_B_C‐3^1^, in total 2 H), 3.04 (dd, 1 H, *J*=4.0/18.0 Hz, H_B_C‐15), 3.17–3.22 (m, HC‐2′, HC‐5′, in total 2 H), 3.23 (triplettoid, 1 H, *J*=9.2 Hz, HC‐4′), 3.27–3.30 (m, 1 H, HC‐3′), 3.60–3.63 (m, 1 H, H_B_C‐3^2^), 3.73 (s, 3 H, H_3_C‐8^5^), 3.89–3.94 (m, HC‐1′) 3.94/ 4.08 (A/B‐system, *J*_AB_=16.4, H_2_C‐5), 3.97 (doublettoid, *J*=6.8 Hz, H_A_C‐6′), superimposed by 4.06–4.15 (m, H_B_C‐6′)—in total 5 H, 4.50–4.55 (m, 1 H, HC‐16), 4.54 (s, ca. 0.5 H, HC‐8^2^), 5.22 (dd, 1 H, *J*=2.0/11.7 Hz, H_A_C‐18^2^), 6.23 (dd, 1 H, *J*=2.1/18.0 Hz, H_B_C‐18^2^), 6.54 (dd, 1 H, *J*=11.6/17.7 Hz, HC‐18^1^), 9.29 ppm (s, 1 H, HC‐20) (see Supporting Information, Figure S7). ^13^C‐NMR data: see Supporting Information, Table S2. ESI‐MS (LCQ): *m*/*z* (%)=811.3 (29, [*M*+Na]^+^); 791.2 (14), 790.1 (46), 789.1 (100, C_41_H_49_O_12_N_4_^+^, [*M*+H]^+^). Fluorescence MeOH (*c*=4.4×10^−6^ m): emission spectrum (*λ*_ex_=357 nm): *λ*_max_=440 nm; excitation spectrum (*λ*_em_=438 nm): *λ*_max_ (rel. int.)=357 (0.97), 320 (1.0) nm.

***Vv‐*** **NCC‐57**: UV/Vis (online, 50 mm aq. phosphate buffer pH7: MeOH ca. 1:1) (rel. *ϵ*): *λ*_max_=314 (1.00), 245sh (1.15), 216 (1.76). ESI‐MS: *m*/*z* (%)=1289.6 (5, [2 *M*+H]^+^); 705.2 (8, [*M*−H+K+Na]^+^); 683.2 (54, [*M*+K]^+^); 667.3 (49, [*M*+Na]^+^); 647.3 (8), 646.3 (39), 645.3 (100, C_35_H_41_N_4_O_8_^+^, [*M*+H]^+^); 613.3 (15, [*M*‐CH_4_O+H]^+^); 522.2 (6, [*M*‐C_7_H_9_NO+H]^+^) (see Supporting Information, Figure S2).

***Vv*** **‐DYCC‐63**: UV/Vis (online, 50 mm aq. phosphate buffer pH7: MeOH ca. 1:1) (rel. *ϵ*): *λ*_max_=426 (1.00), 245sh (0.77), 216 (1.51). ESI‐MS: *m*/*z* (%)=1299.5 (13, [2 *M*+K]^+^); 1283.5 (39, [2 *M*+Na]^+^); 1261.5 (27, [2 *M*+H]^+^); 669.2 (42, [*M*+K]^+^); 653.3 (77, [*M*+Na]^+^); 633.3 (8), 632.3 (39), 631.3 (100, C_34_H_39_N_4_O_8_^+^, [*M*+H]^+^); 599.2 (27, [*M*−CH_4_O+H]^+^) (see Supporting Information, Figure S3).

## Conflict of interest

The authors declare no conflict of interest.

## Supporting information

As a service to our authors and readers, this journal provides supporting information supplied by the authors. Such materials are peer reviewed and may be re‐organized for online delivery, but are not copy‐edited or typeset. Technical support issues arising from supporting information (other than missing files) should be addressed to the authors.

Supplementary
