# Supplementary material for: Novel Types of Hypermodified Fluorescent Phyllobilins from Breakdown of Chlorophyll in Senescent Leaves of Grapevine (Vitis vinifera)
Source: Chemistry. 2018 Oct 30;24(65):17268–79. doi: 10.1002/chem.201803128 (PMC6282590; doi:10.1002/chem.201803128)
Supplement: Supplementary file 1 — Supplementary [file CHEM-24-17268-s001.pdf]

CHEMISTRY
A European Journal

Supporting Information

Novel Types of Hypermodified Fluorescent Phyllobilins from Breakdown of Chlorophyll in Senescent Leaves of Grapevine (*Vitis vinifera*)

Theresia Erhart+,[a] Cecilia Mittelberger+,[b] Xiujun Liu+,[a, c] Maren Podewitz+,[d] Chengjie Li+,[a, e] Gerhard Scherzer,[a] Gertrud Stoll,[b] Josep Valls,[b, f] Peter Robatscher,[b] Klaus R. Liedl,[d] Michael Oberhuber,[b] and Bernhard Kräutler*[a]

chem_201803128_sm_miscellaneous_information.pdf

Supporting Information

General

Chemicals.

HPLC grade methanol (MeOH) was from VWR chemicals (F), potassium dihydrogen phosphate *puriss. p.a.* and potassium phosphate dibasic-anhydrous *puriss. p.a.*, were from Sigma Aldrich (St. Louis, USA), ultrapure water (18 MΩcm^{-1}) from a Millipore apparatus.

HPLC.

Dionex UltiMate 3000 HPLC system, UltiMate 3000 pump, UltiMate 3000 diode array detector and RF2000 fluorescence detector, 200 µl injection loop. Phenomenex Hyperclone ODS 5 µm 250 x 4.6 mm i.d. column protected with a Phenomenex ODS 4x3 mm i.d. pre-column; flow-rate 0.5 ml min^{-1}. Solvent A: 50 mM aq. potassium phosphate (pH 7.0), solvent B: MeOH; solvent composition: (A/B) 0-5 min: 80/20; 5-55 min: 80/20 to 30/70; 55-60 min: 30/70 to 0/100; 60-70 min: 0/100; 70-75 min: 0/100 to 80/20. Data were collected and processed with Chromeleon V6.80.

Spectroscopy.

CD: Jasco J715, $\lambda_{min/max}$ [nm] ($\Delta\varepsilon$); NMR spectroscopy: BrukerUltraShield Avance II+600 MHz or Varian Unity Inova 500 MHz or Bruker Avance 4 Neo 700 MHz spectrometers; δ [ppm], J [Hz], ($\delta(C^{1}\mathit{H}D_2OD)$ 3.31 ppm, and $\delta(^{13}\mathit{C}D_3OD)$ 49.0 ppm,[1] ^{13}C-signal assignments from HSQC and HMBC spectra; mass spectrometry: Thermo LTQ Orbitrap, electrospray ionization (ESI) source, (+)-ion mode, 4.5 kV spray voltage, m/z (rel. abundance, type of ion).

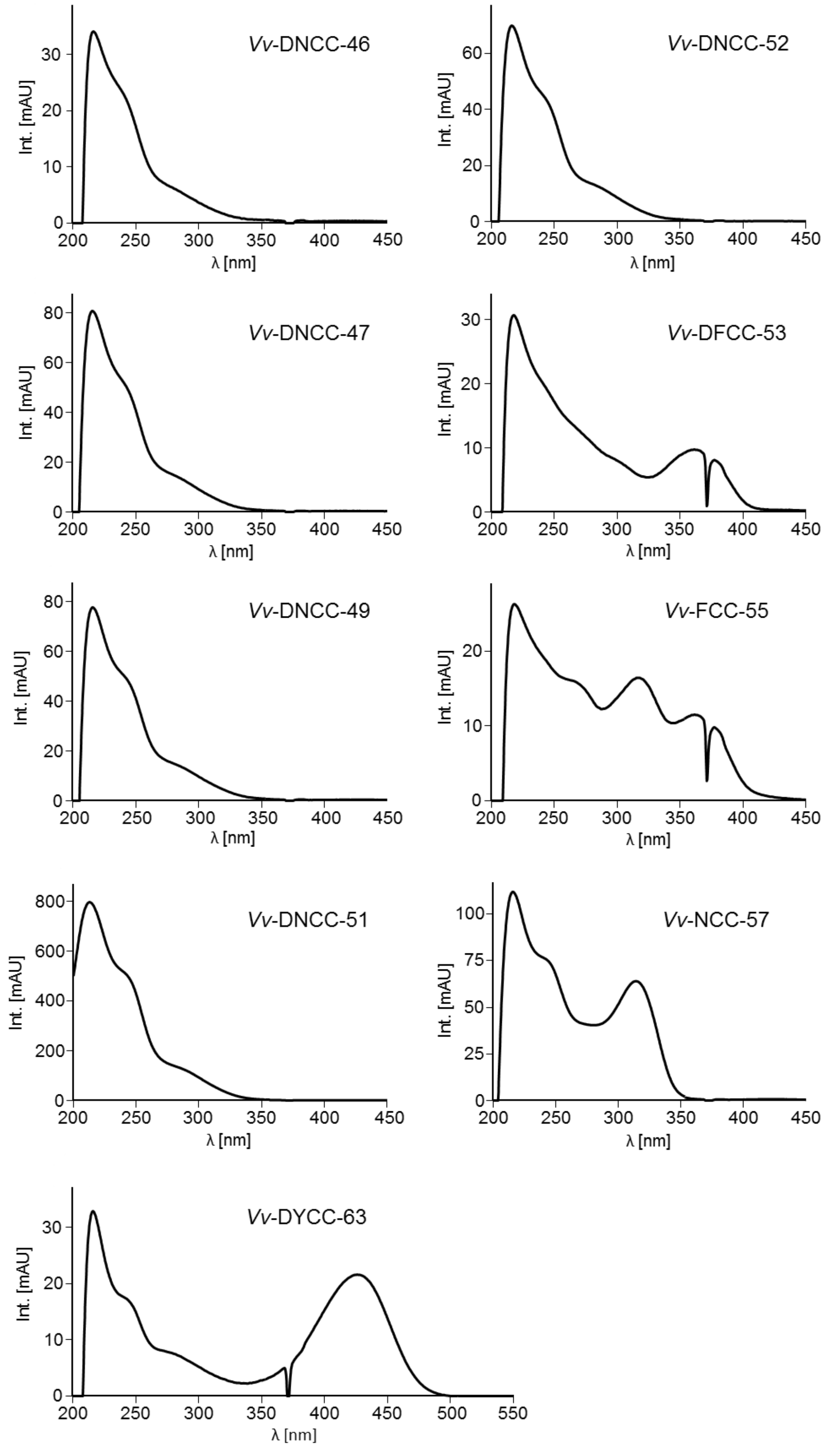

Figure S1. Online UV/Vis-spectra classifying the PB-fractions observed by HPLC-analysis of an extract of senescent *V. vinifera* leaves (see Figure 1 and main text for details of HPLC-experiments).

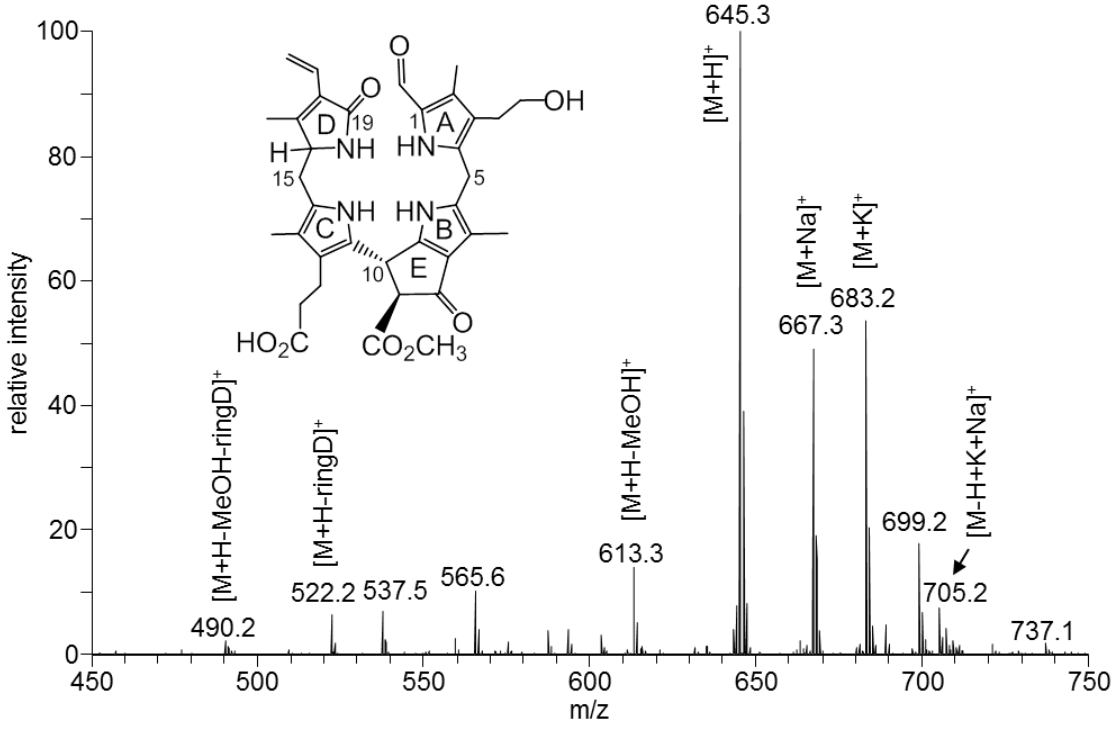

Figure S2. ESI-MS of ***Vv*-NCC-57** (positive ion detection and proposed peak assignment).

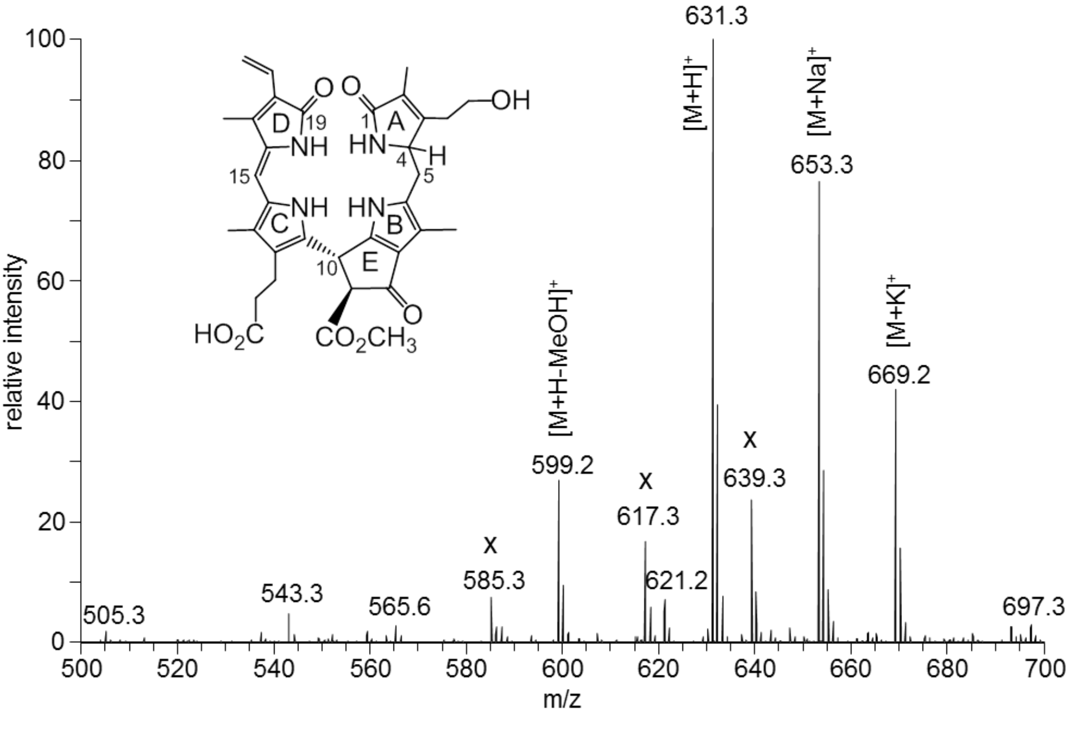

Figure S3. ESI-MS of ***Vv*-DYCC-63** (positive ion detection and proposed peak assignment, signals marked by x are due to an unidentified impurity).

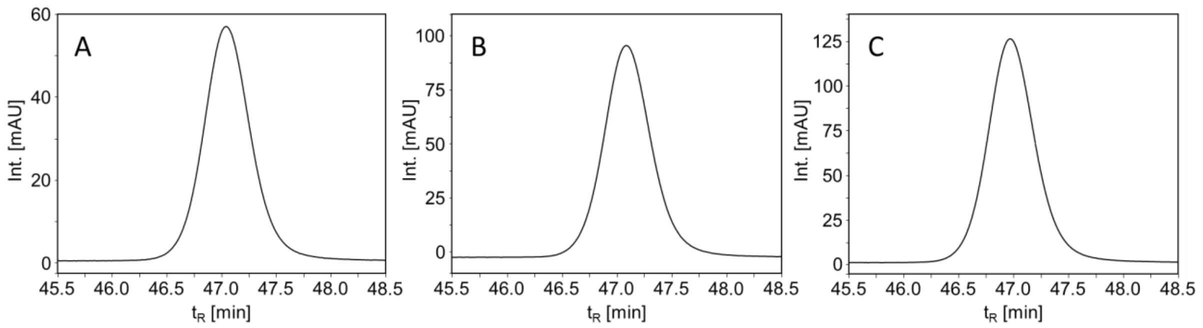

Figure S4. HPLC-analytical traces of A: ***Vv*-NCC-57**, B: ***Cj*-NCC-1**, C: of a co-injection of a roughly 1:1 mixture of ***Vv*-NCC-57** and ***Cj*-NCC-1**.

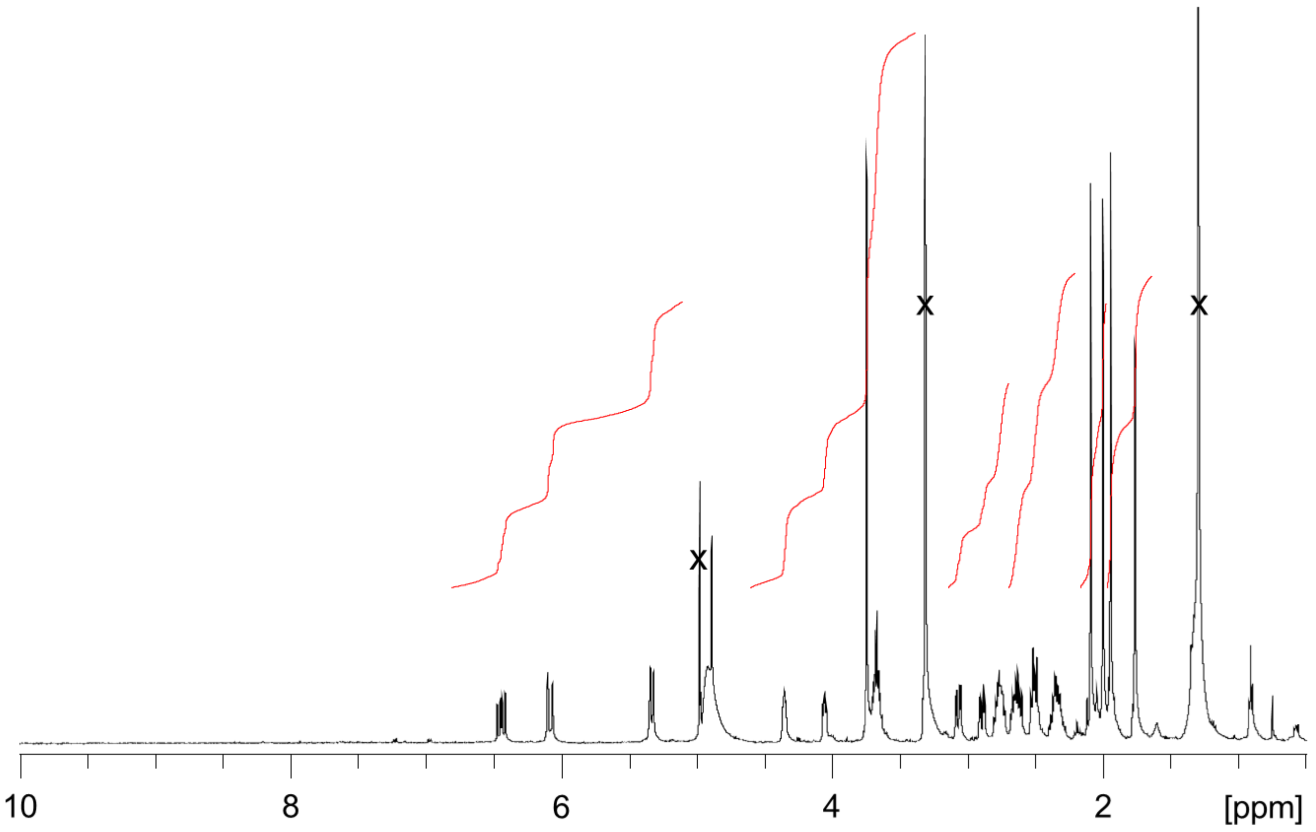

Figure S5. 500 MHz ^{1}H-NMR-spectrum of ***Vv*-DNCC-51** (CD_3OD, 12 °C, x marks residual solvent signals and impurities).

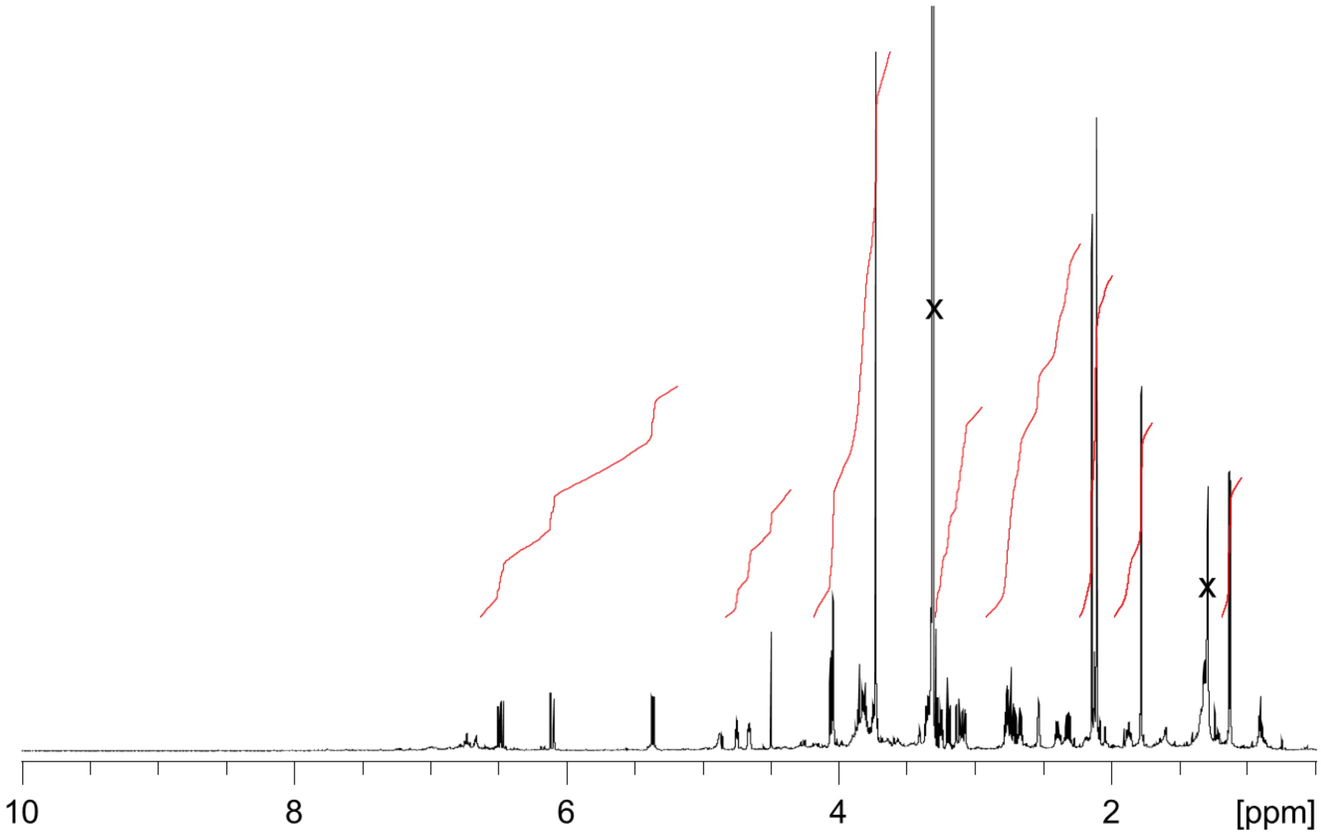

Figure S6. 700 MHz ^{1}H-NMR-spectrum of ***Vv*-DFCC-53** (CD_3OD, 25 °C, x marks residual solvent signals and impurities).

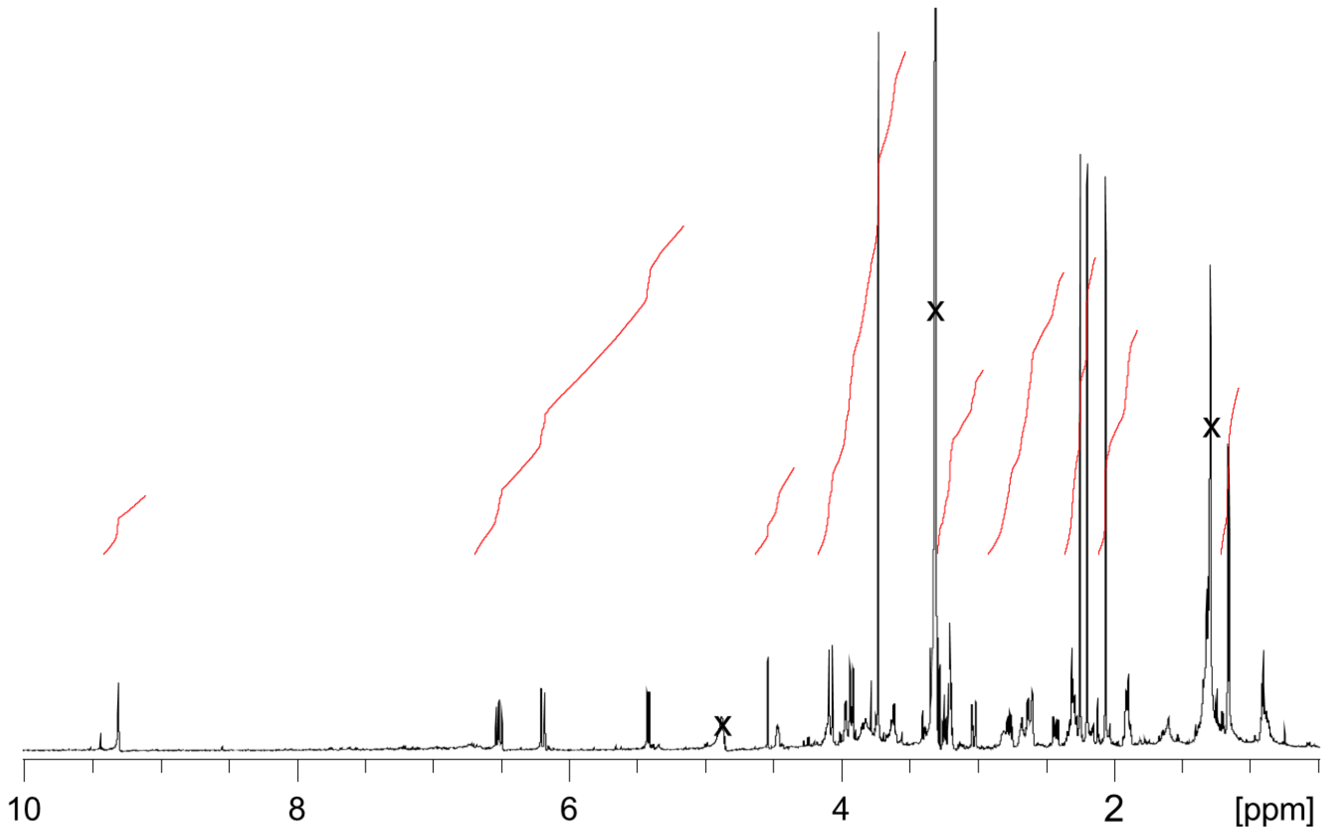

Figure S7. 700 MHz ^{1}H-NMR-spectrum of ***Vv*-FCC-55** (25 °C, CD_3OD, x marks residual solvent signals and impurities).

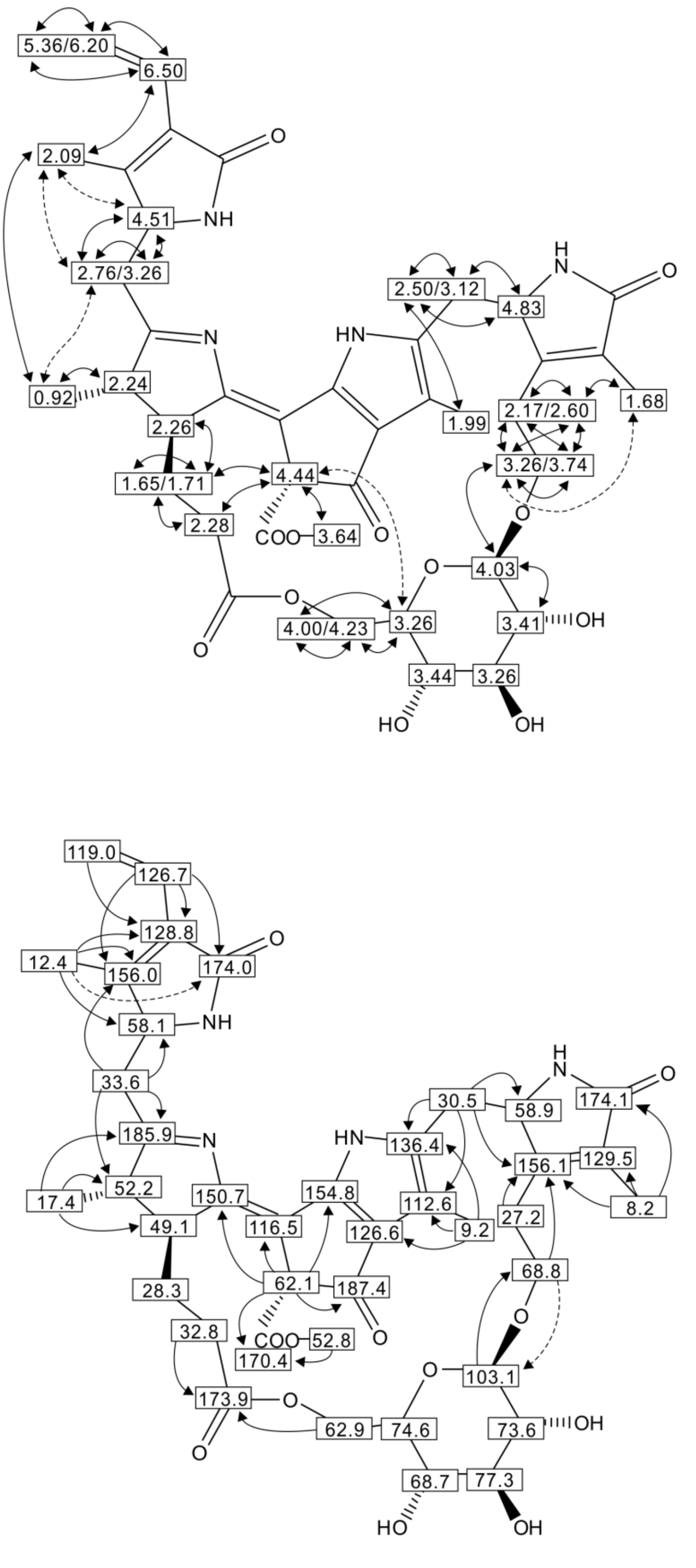

Figure S8. Graphical analysis of the NMR-data of ***Vv*-DFCC-53** (500 MHz, CD_3CN, 0°C) from homonuclear $^1H,^1H$-COSY and $^1H,^1H$-ROESY-spectra (top; full and dotted arrows refer to strong and weaker ROESY-correlations, respectively) and from heteronuclear $^1H,^{13}C$-HSQC- and HMBC-spectra (bottom; arrows indicate HMBC-correlations).

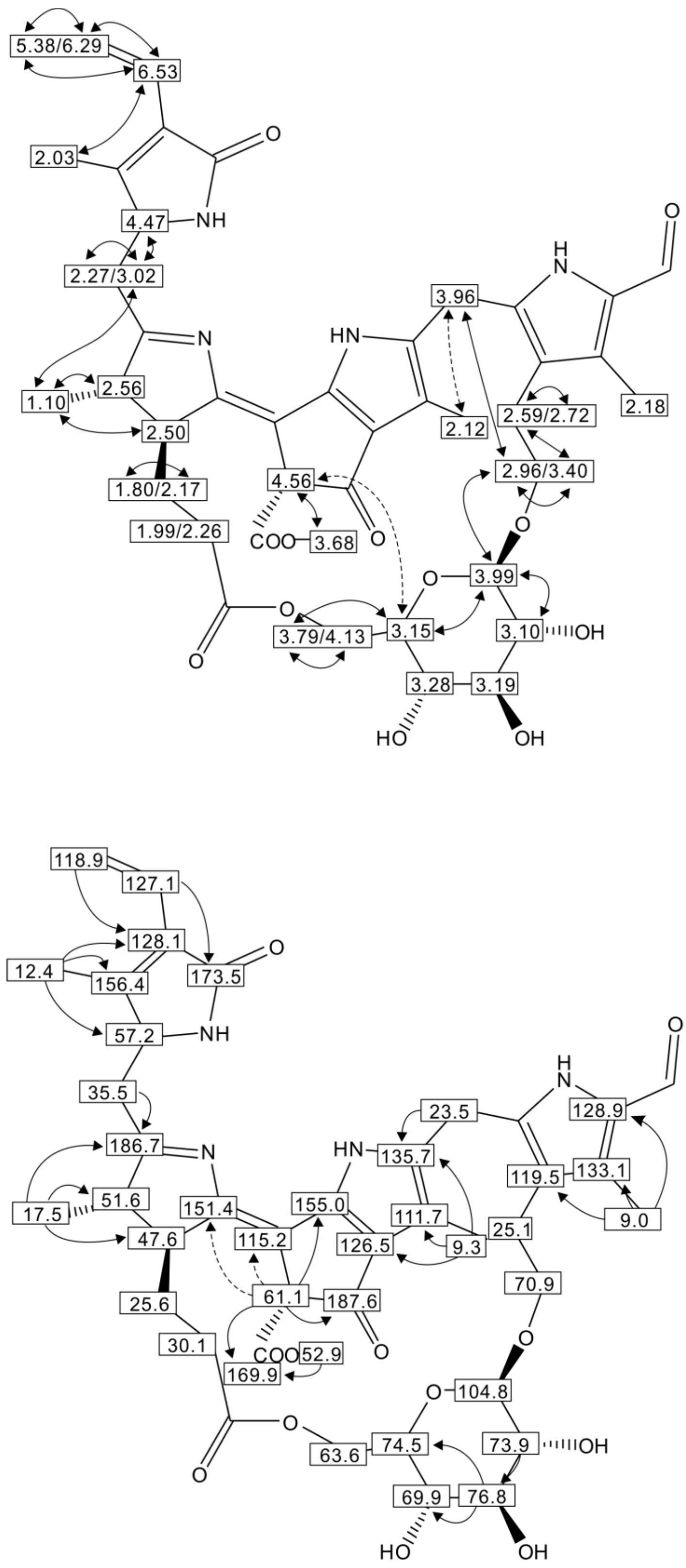

Figure S9. Graphical analysis of the NMR-data of ***Vv*-FCC-55** (600 MHz, CD_3CN, 0°C) from homonuclear $^{1}H,^{1}H$-COSY and $^{1}H,^{1}H$-ROESY-spectra (top; full and dotted arrows refer to strong and weaker ROESY-correlations, respectively) and from heteronuclear $^{1}H,^{13}C$-HSQC- and HMBC-spectra (bottom; arrows indicate HMBC-correlations).

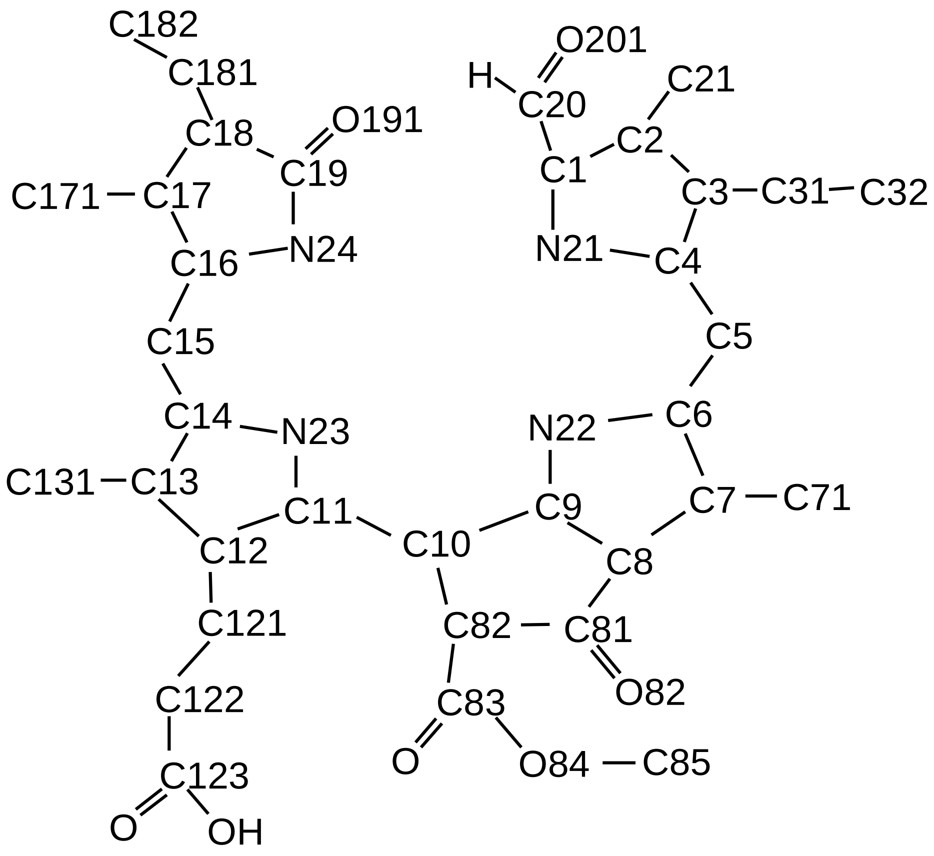

Figure S10. Atom numbering of phyllobilins (represented by an FCC), used for signal assignments in NMR-spectra. Note: in DNCCs and DFCCs the formyl group at C1 is replaced by a carbonyl group.

Table 1. Chemical shift values of ^{1}H- and ^{13}C signals in 500 MHz-NMR spectra (CD_3OD, 12 °C) of ***Vv*-DNCC-51** and comparison with those of its epimer *At*$_{Mes}$-DNCC-38 [2]

	***Vv*-DNCC-51** **δ^{13}C**	***Vv*-DNCC-51** **δ^{1}H(ppm)/J(Hz)**	***At*$_{Mes}$-DNCC-38** **δ^{13}C**	***At*$_{Mes}$-DNCC-38** **δ^{1}H(ppm)/J(Hz)**
**C1**	175.2		176.2	
**C2**	129.4		130.5	
**C2^1**	8.30	1.76, s	8.0	1.77, s
**C3**	154.6		155.6	
**C3^1**	30.8	2.49/2.78	31.0	2.50, m<br>2.79, m
**C3^2**	60.1	3.67, m	60.8	3.70, m
**C4**	59.2	4.35, triplettoid, J~5.6	60.5	4.35, dd, *J* ~ 4.7/8.7
**C5**	28.6	2.62<br>3.07, dd, *J* = 5.0/14.8	30.0	2.54, dd, *J* = 8.8/14.7<br>3.08, dd, *J* = 4.6/14.8
**C6**	132.8		134.4	
**C7**	111.4		112.5	
**C7^1**	9.3	2.09, s	9.50	2.11, s
**C8**	124.3		125.6	
**C8^1**				
**C8^2**	66.3		67.5	
**C8^3**	170.5		171.4	
**C8^5**	52.5	3.74, s	52.3	3.75, s
**C9**	160.0		160.9	
**C10**	37.0	4.89, s	36.7	4.93, s
**C11**	122.9		123.9	
**C12**	119.3		120.3	
**C12^1**	22.0	2.66/2.74	21.2	2.64, m<br>2.72, m
**C12^2**	39.8	2.34, m	38.4	2.36, m
**C12^3**	180.4		180.0	
**C13**	114.0		114.9	
**C13^1**	8.3	1.94, s	8.9	1.96, s
**C14**	122.9		124.2	
**C15**	30.1	2.51<br>2.89, dd, *J* = 5.0/14.6	30.2	2.45, dd, *J* =9.1/14.5<br>2.91, dd, *J* = 4.8/14.5
**C16**	60.7	4.05, dd, *J* = 4.9/8.5	61.3	4.09, dd, *J* = 4.8/9.1
**C17**	155.4		156.5	
**C17^1**	12.4	2.00, s, 3H	12.1	1.99, s
**C18**	127.4		128.3	
**C18^1**	126.9	6.44, dd, *J* = 11.7/17.8	126.9	6.44, dd, *J* = 11.8/17.8
**C18^2**	118.7	5.33, dd, *J* = 2.2/11.7<br>6.08, dd, *J* = 2.2/17.8	118.7	5.32, dd, *J* = 2.0/11.8<br>6.08, dd, *J* = 2.0/17.8
**C19**	174.6		174.2	

Table 2. Chemical shift values of ^{1}H- and ^{13}C signals in 600 MHz-NMR spectra (CD_3OD, 0 °C) of ***Vv*-DFCC-53** and ***Vv*-FCC-55** and their assignments (*C8^{2} is partially deuterated).

	**δ^{13}C (*Vv*-DFCC-53)**	**δ^{1}H/J (*Vv*-DFCC-53)**	**δ^{13}C (*Vv*-FCC-55)**	**δ^{1}H/J (*Vv*-FCC-55)**
**C1**	175.9		129.5	
**C2**	130.4		135.5	
**C2^{1}**	8.1	1.78, s	8.9	2.25, s
**C3**	156.6		120.2	
**C3^{1}**	27.7	2.37, m<br>2.75	25.4	2.67, m<br>2.76
**C3^{2}**	68.6	3.33<br>3.75, m	70.6	2.75<br>3.58, m
**C4**	59.5	4.67, m	137.2	
**C5**	30.1	2.70, dd, *J* = 9.4/14.6<br>3.09, dd, *J* = 4.9/14.6	23.5	3.94, dd, J_{AB} = 16.4<br>4.08, dd, J_{AB} = 16.4
**C6**	136.4		136.4	
**C7**	113.7		112.5	
**C7^{1}**	9.3	2.11, s	9.2	2.20, s
**C8**	127.0		127.0	
**C8^{1}**	189.6		-	
**C8^{2}**	61.9	4.52, s*	61.2	4.54, s*
**C8^{3}**	170.7		170.7	
**C8^{5}**	52.7	3.73, s	52.9	3.73, s
**C9**	156.1		-	
**C10**	116.7		-	
**C11**	152.6			
**C12**	48.1	2.52, m	48.0	2.59, m
**C12^{1}**	27.5	1.87, m<br>2.09	25.9	1.89<br>2.30
**C12^{2}**	30.8	2.14<br>2.32, m	29.8	1.90<br>2.31
**C12^{3}**	174.9		174.9	
**C13**	53.3	2.66, q, *J* = 7.2/14.4	52.3	2.64, dd, *J* = 2.6/7.3
**C13^{1}**	17.7	1.12, d, *J* = 7.3	17.7	1.17, d, *J* = 7.3
**C14**	187.0		187.0	
**C15**	34.2	2.76<br>3.14, dd, *J* = 5.2/17.0	35.6	2.42, dd, *J* = 8.9/18.0<br>3.04, dd, *J* = 4.0/18.0
**C16**	58.4	4.80, t, *J* = 5.6/5.6	57.9	4.53, m
**C17**	156.6		157.0	
**C17^{1}**	12.4	2.15, s	12.5	2.07, s
**C18**	129.6		129.0	
**C18^{1}**	126.6	6.50, dd, *J* = 11.7/17.7	126.9	6.54, dd, *J* = 11.6/17.7
**C18^{2}**	119.2	5.37, dd, *J* = 2.2/11.7<br>6.14, dd, *J* = 2.2/17.7	119.4	5.22, dd, *J* = 2.0/11.7<br>6.23, dd, *J* = 2.1/18.0
**C19**	174.8		174.9	
**C20**			177.6	9.29, s
**C1´**	103.8	4.07, d, *J* = 7.8	104.9	3.97, duplettoid, J ~ 6.8
**C2´**	74.6	3.20, m	74.9	3.20
**C3´**	77.7	3.29	77.4	3.29, m
**C4´**	71.3	3.33	71.3	3.23, triplettoid, J ~ 9.2
**C5´**	74.8	3.25	74.9	3.20
**C6´**	64.8	4.05, apparent d	65.0	3.91 / 4.14

Table 3. Comparison of ^{1}H- and ^{13}C-NMR data of ***Vv*-FCC-55** (500/600 MHz) and of *Ug*-NCC-53 [3] (600 MHz) in CD_3OD (*C8^2 is partially deuterated)

	**δ^{13}C (*Vv*-FCC-55)**	**δ^{1}H/J (*Vv*-FCC-55)**	**δ^{13}C (*Ug*-NCC-53)**	**δ^{1}H/J (*Ug*-NCC-53)**
**C1**	129.5		129.2	
**C2**	135.5		135.5	
**C2^1**	8.9	2.25, s	8.8	2.27, s
**C3**	120.2		121.3	
**C3^1**	25.4	2.67, m<br>2.76	25.4	2.70, t, *J* = 6.7
**C3^2**	70.6	2.75<br>3.58, m	71.6	3.45-3.53, m<br>3.63-3.69, m
**C4**	137.2		138.3	
**C5**	23.5	3.94, dd, J_{AB} = 16.4<br>4.08, dd, J_{AB} = 16.4	23.6	3.91, d, *J* = 17.0<br>3.97, dd, *J* = 17.0
**C6**	136.4		134.2	
**C7**	112.5		112.5	
**C7^1**	9.2	2.20, s	9.0	2.13, s
**C8**	127.0		125.8	
**C8^2**	61.2	4.54, s*	68.1	3.78, d, *J* = 3.1
**C8^3**	170.7		171.6	
**C8^5**	52.9	3.73, s	52.7	3.76, s
**C9**			160.9	
**C10**			38.1	4.88
**C11**			124.5	
**C12**	48.0	2.59, m	119.8	
**C12^1**	25.9	1.89<br>2.30	19.8	2.40-2.47, m<br>2.60-2.68, m
**C12^2**	29.8	1.90<br>2.31	36.1	2.31-2.39, m
**C12^3**	174.9		174.4	
**C13**	52.3	2.64, dd, *J* = 2.6/7.3	115.5	
**C13^1**	17.7	1.17, d, *J* = 7.3	9.0	1.89, s
**C14**	187.0		123.9	
**C15**	35.6	2.42, dd, *J* = 8.9/18.0<br>3.04, dd, *J* = 4.0/18.0	30.1	2.50, dd, *J* = 8.6/14.6<br>2.89, dd, *J* = 5.1/14.6
**C16**	57.9	4.53, m	61.5	4.09, dd, *J* = 5.1/8.6
**C17**	157.0		156.6	
**C17^1**	12.5	2.07, s	12.5	2.01, s
**C18**	129.0		128.6	
**C18^1**	126.9	6.54, dd, *J* = 11.6/17.7	126.8	6.45, dd, *J* = 11.7/17.7
**C18^2**	119.4	5.22, dd, *J* = 2.0/11.7<br>6.23, dd, *J* = 2.1/18.0	119.0	5.35, dd, *J* = 2.2/11.7<br>6.10, dd, *J* = 2.2/17.7
**C19**	174.9		174.6	
**C20**	177.6	9.29, s	177.5	9.32, s
**C1´**	104.9	3.97, duplettoid, J ~ 6.8	104.6	4.23, d, *J* = 7.9
**C2´**	74.9	3.20	74.8	3.13, dd, *J* = 7.9/9.1
**C3´**	77.4	3.29	77.8	3.35, triplettoid, *J* ~ 9.5
**C4´**	71.3	3.23, triplettoid, J ~ 9.2	71.6	3.25, triplettoid, *J* ~ 9.5
**C5´**	74.9	3.20	75.2	3.45-3.53, m, *J* = 2.3/6.7
**C6´**	65.0	3.91 / 4.14	64.7	3.97 (d, *J* = 17.0) / 4.56, m

Table 4. Comparison of ^{1}H- and ^{13}C signals in 600 MHz-NMR spectra of ***Vv*-DFCC-53** at 0 °C in CD_3OD (2 left columns, *C8^{2} partially deuterated) and in 500 MHz-NMR spectra in CD_3CN (2 right columns).

	**δ^{13}C (*Vv*-DFCC-53)**	**δ^{1}H/J (*Vv*-DFCC-53)**	**δ^{13}C (*Vv*-DFCC-53)**	**δ^{1}H/J (*Vv*-DFCC-53)**
**C1**	175.9		174.1	
**C2**	130.4		129.5	
**C2^{1}**	8.1	1.78, s	8.2	1.68, s
**C3**	156.6		156.1	
**C3^{1}**	27.7	2.37, m<br>2.75	27.2	2.17, m<br>2.60, dt, *J* = 3.5 / 14.2
**C3^{2}**	68.6	3.33<br>3.75, m	68.8	3.26, m<br>3.76, m
**C4**	59.5	4.67, m	58.9	4.83, m
**C5**	30.1	2.70, dd, *J* = 9.4/14.6<br>3.09, dd, *J* = 4.9/14.6	30.5	2.50, dd, *J* = 10.2 / 14.3<br>3.12, dd, *J* = 4.7 / 14.3
**C6**	136.4		136.4	
**C7**	113.7		112.6	
**C7^{1}**	9.3	2.11, s	9.2	1.99, s
**C8**	127.0		126.6	
**C8^{1}**	189.6		187.4	
**C8^{2}**	61.9	4.52, s*	62.1	4.44, s
**C8^{3}**	170.7		170.4	
**C8^{5}**	52.7	3.73, s	52.8	3.64, s
**C9**	156.1		154.6	
**C10**	116.7		116.5	
**C11**	152.6		150.7	
**C12**	48.1	2.52, m	**49.1**	**2.26, m**
**C12^{1}**	27.5	1.87, m<br>2.09	28.3	1.65, m<br>1.71, m
**C12^{2}**	30.8	2.14<br>2.32, m	32.8	2.28, m
**C12^{3}**	174.9		173.9	
**C13**	53.3	2.66, q, *J* = 7.2/14.4	52.2	**2.24, m**
**C13^{1}**	17.7	1.12, d, *J* = 7.3	17.4	0.92, d, *J* = 7.3
**C14**	187.0		185.9	
**C15**	34.2	2.76<br>3.14, dd, *J* = 5.2/17.0	33.6	2.76, dd, *J* = 3.5 / 15.9<br>3.26, m
**C16**	58.4	4.80, t, *J* = 5.6/5.6	58.1	4.51, m
**C17**	156.6		156.0	
**C17^{1}**	12.4	2.15, s	12.4	2.09, s
**C18**	129.6		128.8	
**C18^{1}**	126.6	6.50, dd, *J* = 11.7/17.7	126.7	6.50, dd, *J* = 11.6 / 17.7
**C18^{2}**	119.2	5.37, dd, *J* = 2.2/11.7<br>6.14, dd, *J* = 2.2/17.7	119.0	5.36, dd, *J* = 2.4 / 11.6<br>6.20, dd, *J* = 2.4 / 17.7
**C19**	174.8		174.0	
**C20**				
**C1´**	103.8	4.07, d, *J* = 7.8	103.1	4.03, d, *J* = 7.8
**C2´**	74.6	3.20, m	73.6	3.41, m
**C3´**	77.7	3.29	77.3	3.26, m
**C4´**	71.3	3.33	68.7	3.44, m
**C5´**	74.8	3.25	74.6	3.26, m
**C6´**	64.8	4.05, apparent d	62.9	4.23 d, *J* = 11.0<br>4.00, dd, *J* = 3.6 / 11.0
**NH#**				8.55; 10.17

broad signals and tentative assignments due to lack of homo- and hetero-nuclear correlations

Table 5. Comparison of ^{1}H- and ^{13}C signals in 600 MHz-NMR spectra of ***Vv*-FCC-55** at 0 °C in CD_3OD (two left columns,*C8^2 is partially deuterated) and in CD_3CN (two right columns).

	**δ^{13}C (*Vv*-FCC-55)**	**δ^{1}H/J (*Vv*-FCC-55)**	**δ^{13}C (*Vv*-FCC-55)**	**δ^{1}H/J (*Vv*-FCC-55)**
**C1**	129.5		128.9	
**C2**	135.5		133.1	
**C2^1**	8.9	2.25, s	9.0	2.18, s
**C3**	120.2		119.5	
**C3^1**	25.4	2.67, m<br>2.76	25.1	2.59, m<br>2.72, m
**C3^2**	70.6	2.75<br>3.58, m	70.9	2.96, m<br>3.40, m
**C4**	137.2			
**C5**	23.5	3.94, dd, J_{AB} = 16.4<br>4.08, dd, J_{AB} = 16.4	23.5	3.96, d, *J* = 6.2
**C6**	136.4		135.7	
**C7**	112.5		111.7	
**C7^1**	9.2	2.20, s	9.3	2.12, s
**C8**	127.0		126.5	
**C8^2**	61.2	4.54, s*	61.1	4.56, s
**C8^3**	170.7		169.9	
**C8^5**	52.9	3.73, s	52.9	3.68, s
**C9**			155.0	
**C10**			115.2	
**C11**			151.4	
**C12**	48.0	2.59, m	47.6	2.50, m
**C12^1**	25.9	1.89<br>2.30	25.6	1.80, m<br>2.17, m
**C12^2**	29.8	1.90<br>2.31	30.1	1.99, m<br>2.16, m
**C12^3**	174.9			
**C13**	52.3	2.64, dd, *J* = 2.6/7.3	51.6	2.56, m
**C13^1**	17.7	1.17, d, *J* = 7.3	17.5	1.10, d, *J* = 7.3
**C14**	187.0		186.7	
**C15**	35.6	2.42, dd, *J* = 8.9/18.0<br>3.04, dd, *J* = 4.0/18.0	35.5	2.27, m<br>3.02,dd, *J* = 2.7 / 18.4
**C16**	57.9	4.53, m	57.2	4.47, d, *J* = 9.5
**C17**	157.0		156.4	
**C17^1**	12.5	2.07, s	12.4	2.03, s
**C18**	129.0		128.1	
**C18^1**	126.9	6.54, dd, *J* = 11.6/17.7	127.1	6.53, dd, *J* = 11.6, 17.7
**C18^2**	119.4	5.22, dd, *J* = 2.0/11.7<br>6.23, dd, *J* = 2.1/18.0	118.9	5.38, dd, *J* = 2.2 / 11.6<br>6.29, dd, *J* = 2.2 / 17.7
**C19**	174.9		173.5	
**C20**	177.6	9.29, s		9.29#
**C1´**	104.9	3.97, duplettoid, J ~ 6.8	104.8	3.99, d, *J* = 7.6
**C2´**	74.9	3.20	73.9	3.10, t, *J* = 8.0
**C3´**	77.4	3.29	76.8	3.19, t, *J* = 9.0
**C4´**	71.3	3.23, triplettoid, J ~ 9.2	69.9	3.28, m
**C5´**	74.9	3.20	74.5	3.15, m
C6´	65.0	3.91 / 4.14	63.6	3.79, m<br>4.14, d, *J* = 11.4
NH#				9.17#; 10.01

broad signals and tentative assignments due to lack of homo- and hetero-nuclear correlations

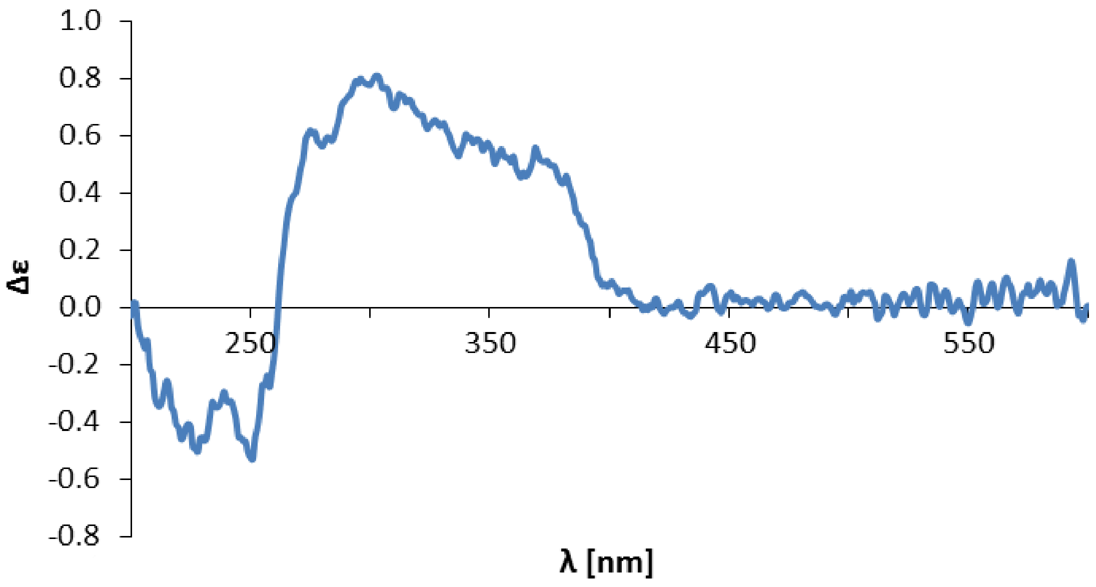

Figure S11. CD spectrum of ***Vv*-DFCC-53** (MeOH, c = $6.6*10^{-5}$ M)

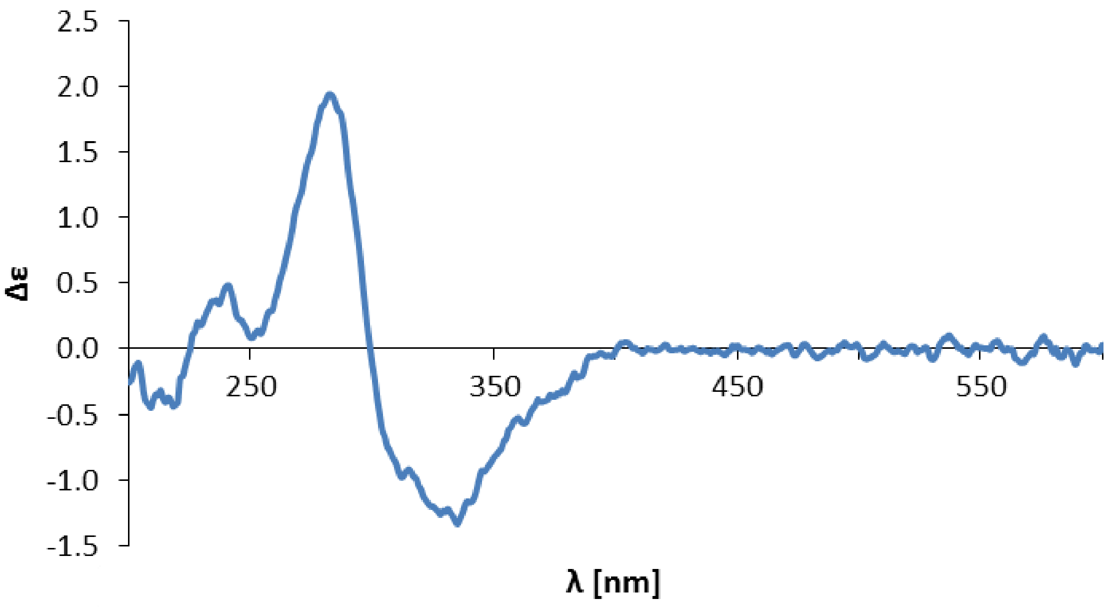

Figure S12. CD spectrum of ***Vv*-FCC-55** (MeOH, c = $5.3*10^{-5}$ M)

Quantum Chemical Investigation.

***Vv*-FCC-55**

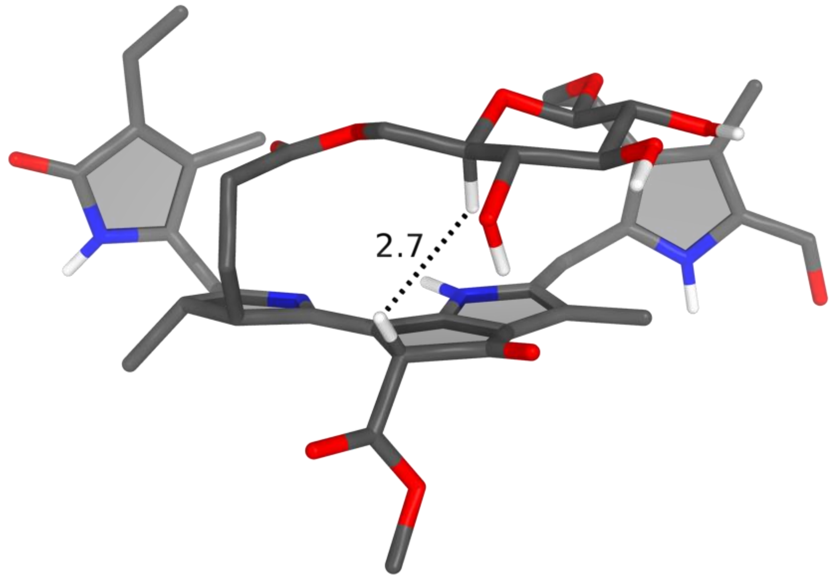

Figure S13. BP86/def2-TZVP optimized gas phase structure of ***Vv*-FCC-55**. The protons HC10 and HC5' are at a calculated mutual distance of 2.7 Å.

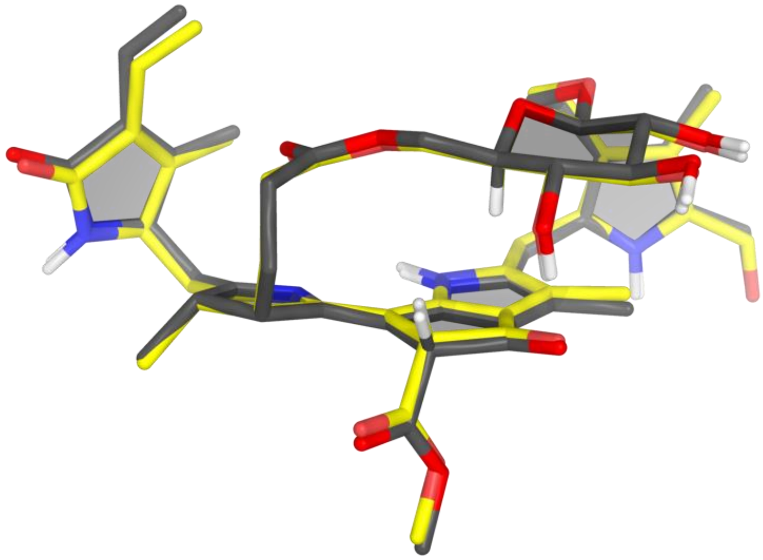

Figure S14. Overlay of the BP86/def2-TZVP and the BP86/def2-TZVP/BJ (including empirical dispersion corrections) structures of ***Vv*-FCC-55**, both are very similar and only show minor differences.

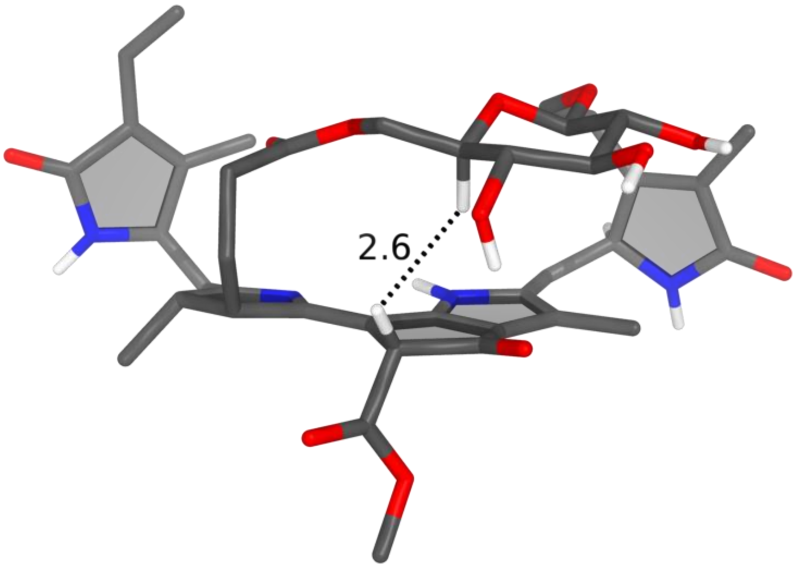

Figure S15. BP86/def2-TZVP optimized gas phase structure of 4*S*-isomer of ***Vv*-DFCC-53**. The protons HC10 and HC5’ are at a distance of 2.6 Å.

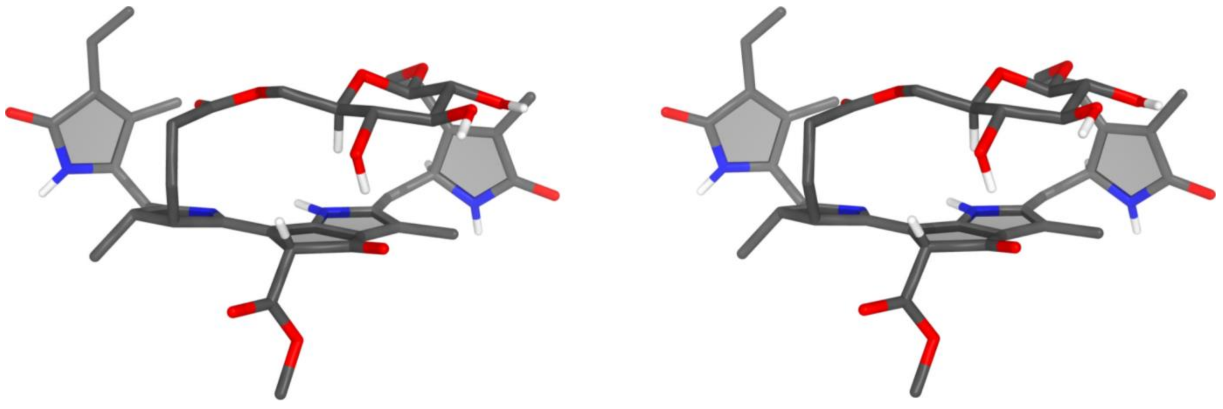

Figure S16. Stereo-projection of the BP86/def2-TZVP optimized gas phase structure of 4*S*-isomer of ***Vv*-DFCC-53**.

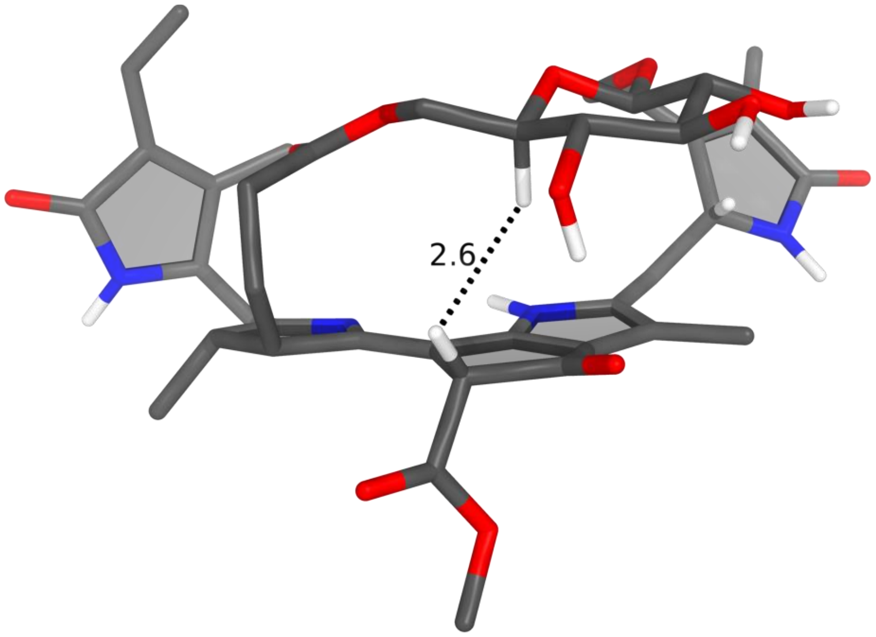

Figure S17. BP86/def2-TZVP optimized gas phase structure of 4*R*-isomer of ***Vv*-DFCC-53**. The protons HC10 and HC5' are at a distance of 2.6 Å.

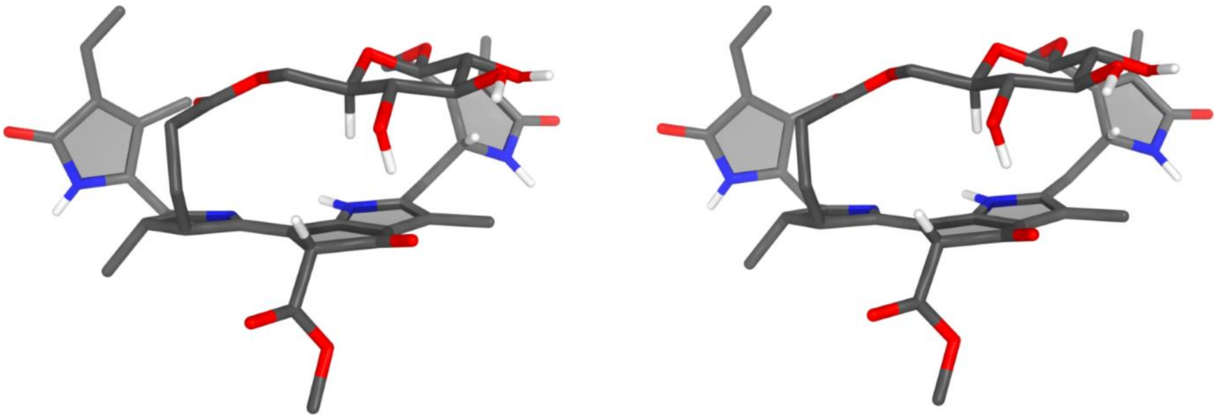

Figure S18. Stereo-projection of the BP86/def2-TZVP optimized gas phase structure of 4*R*-isomer of ***Vv*-DFCC-53**.

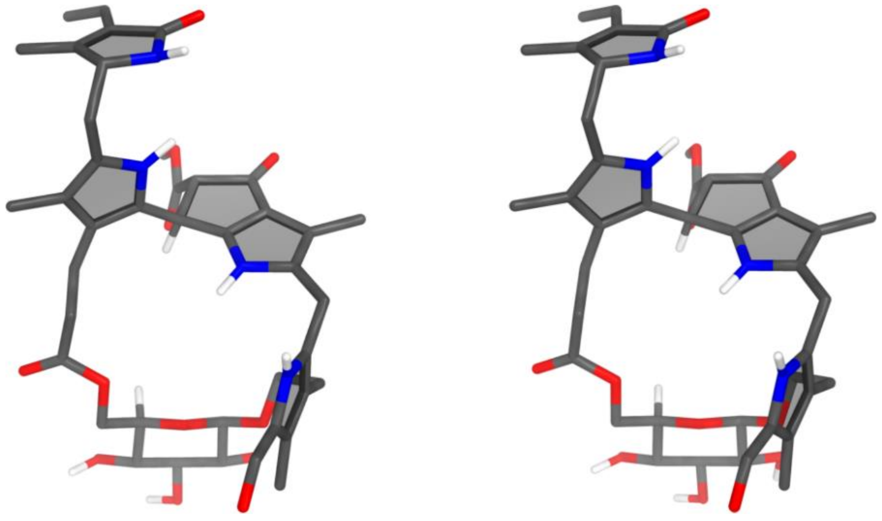

Figure S19. Stereo-projection of the BP86/def2-TZVP optimized gas phase structure of ***Ug*-NCC-53**. Its conformation is derived from earlier molecular dynamics simulations. [3]

References

[1] H. E. Gottlieb, V. Kotlyar and A. Nudelman, *J. Org. Chem.* **1997**, *62*, 7512-7515.
[2] I. Süßenbacher, B. Christ, S. Hörtensteiner and B. Kräutler, *Chem. Eur. J.* **2014**, *20*, 87-92.
[3] M. Scherl, T. Müller, C. Kreutz, R. G. Huber, E. Zass, K. R. Liedl and B. Kräutler, *Chem. Eur. J.* **2016**, *22*, 9498-9503.
